# Different Ferromanganese Concretion Morphologies Host Distinct Microbial Communities and Metal Accumulation Patterns in the Baltic Sea

**DOI:** 10.1111/1462-2920.70390

**Published:** 2026-07-23

**Authors:** Renata Majamäki, Joonas Wasiljeff, Lotta Purkamo, Jenni Hultman, Lukas Kohl, Eero Asmala, Pirjo Yli‐Hemminki, Kirsten S. Jørgensen, Johanna Muurinen, Joonas J. Virtasalo

**Affiliations:** ^1^ Geological Survey of Finland (GTK) Espoo Finland; ^2^ Natural Resources Institute Finland (Luke) Helsinki Finland; ^3^ Department of Microbiology University of Helsinki Helsinki Finland; ^4^ Department of Agricultural Sciences University of Helsinki Helsinki Finland; ^5^ Department of Biological and Environmental Science University of Eastern Finland Kuopio Finland; ^6^ Finnish Environment Institute (Syke) Helsinki Finland

**Keywords:** Baltic Sea, brackish water, ferromanganese, geomicrobiology, microbial ecology, nodules, rare‐earth elements, trace elements

## Abstract

Ferromanganese (Fe‐Mn) concretions are porous accumulations of iron and manganese (hydr)oxides. While recent studies suggest that microbes contribute to metal accumulation in Baltic Sea concretions, the detailed composition of microbial communities and their impact on metal enrichment across different concretion morphotypes remain unexplored. We investigated how microbes influence the accumulation and release of trace metals and rare‐earth elements in Fe‐Mn concretions from the Gulf of Finland through 15‐week microcosm incubation experiments with biotic and abiotic treatments, focusing on three main concretion morphotypes: crust, discoidal, and spheroidal. Elemental analysis showed that microbes enhanced metal incorporation in discoidal and spheroidal morphotypes. Characterisation of microbial composition revealed that all three morphologies host distinct communities. Discoidal and spheroidal morphotypes had a higher relative abundance of Gammaproteobacteria and sulfate‐reducing bacteria, and a lower abundance of Entotheonellaeota, compared to crusts. In all morphotypes, the bacterial phylum Pseudomonadota dominated, with several genera of Fe‐ and Mn‐oxidisers and reducers. Fe‐Mn concretions also host communities involved in methane oxidation and nitrogen cycling, consistent with decreased methane and increased nitrous oxide, nitrite, and nitrate concentrations in the microcosms. Our findings underscore that distinct microbial communities are associated with different concretion morphotypes, potentially influencing nutrient and metal cycling on the seafloor.

## Graphical abstract

We investigated how microbes influence the metal accumulation and release in Fe‐Mn concretions from the Gulf of Finland using microcosm incubation experiments. We studied three main concretion morphotypes: crust, discoidal, and spheroidal. Microbes enhanced metal accumulation in discoidal and spheroidal morphotypes. Characterisation of microbial composition revealed that all three morphologies host distinct communities. Discoidal and spheroidal morphotypes had higher relative abundance of Gammaproteobacteria and sulfate‐reducing bacteria, while crusts had higher abundance of Entotheonellaeota.

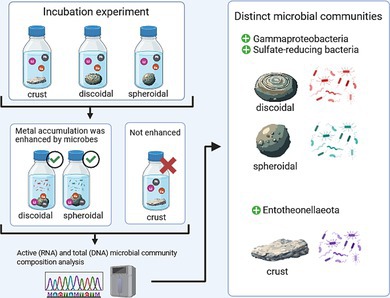

## 1 Introduction

Ferromanganese (Fe‐Mn) concretions are mineral precipitates composed primarily of iron and manganese (hydr)oxides that form on the seafloor. They are common in freshwater and marine shelf environments, as well as oceans, where they are typically called nodules (e.g., Hein and Koschinsky 2014; Margolis and Burns 1976). Fe‐Mn concretions can reach high abundances in shelf seas, such as the brackish‐water Baltic Sea in northern Europe. In Finnish sea areas of the northern Baltic Sea, Fe‐Mn concretion fields are estimated to cover over 11% of the seafloor and occur in patchy patterns (Kaikkonen et al. 2019). Reported abundances of Fe‐Mn concretions range from 18 to 24 kg m^−2^ in the Gulf of Finland, with the highest values observed in the eastern part of the gulf (Glasby et al. 1997). On the seafloor, Fe‐Mn concretions are recognised as a habitat type (HELCOM 2013). They increase habitat complexity by forming porous, hard substrates on otherwise soft seabeds and thereby support a higher diversity of benthic life (Vanreusel et al. 2016). Fe‐Mn concretions have attracted scientific interest, as they provide habitats for diverse microbial communities (Yli‐Hemminki et al. 2014), accumulate phosphorus, economically valuable metals, and rare‐earth elements (REEs) (Zhamoida et al. 2017; Baturin 2009; Hlawatsch, Garbe‐Schönberg, et al. 2002; Wasiljeff, Yu, et al. 2025), and preserve high‐resolution records of past geochemical and oceanographic conditions (Wasiljeff, Lahaye, et al. 2025). Understanding metal accumulation processes and how microbes contribute to them is crucial to elucidating their role in seafloor metal cycling.

The formation of concretions is a biogeochemical process in which dissolved Fe and Mn in seawater and porewater are oxidised and precipitate as Fe and Mn (oxyhydr)oxides on the seafloor. The formation process can occur in seafloor areas where conditions are predominantly oxic and where little or no net sediment deposition occurs. Such conditions are commonly met at locations where silty clays from the previous lacustrine phase of the Baltic Sea Basin are exposed on the seafloor (Winterhalter 2004; Zhamoida et al. 2017). The primary sources of Fe and Mn to the sites of concretion formation are riverine influx (Winterhalter 2004) and the reductive dissolution of Mn and Fe hydroxides in the underlying lacustrine sediments and their upward diffusion in the porewater (Widerlund and Ingri 1996; Virtasalo and Kotilainen 2008), supplemented by benthic efflux and lateral transport from nearby suboxic–anoxic small depressions (Hlawatsch, Neumann, et al. 2002; Winterhalter 2004; Pakhomova et al. 2007). Furthermore, Fe‐Mn concretions are occasionally covered with an organic‐rich ‘fluffy’ mud layer that provides Fe and Mn (Hlawatsch, Neumann, et al. 2002; Wasiljeff, Yu, et al. 2025), and detrital minerals, which are incorporated and redistributed within the Fe‐Mn concretions (Wasiljeff, Yu, et al. 2025). The concretions exhibit various shapes such as flat crusts, small buckshot‐like discoidal, spheroidal, and irregular forms (Zhamoida et al. 2017), but these shapes can be classified into three main morphologies in the Gulf of Finland: crusts, discoidal, and spheroidal, with diameters ranging from a few to several centimetres (Winterhalter 2004; Glasby et al. 1997; Zhamoida et al. 1996; Wasiljeff et al. 2024). The specific morphotype in which a concretion forms is influenced by the hydrodynamics, redox fluctuations and sediment depositional conditions on the seafloor (e.g., Wasiljeff, Yu, et al. 2025; Winterhalter 2004; Zhamoida et al. 1996).

In addition to Fe and Mn, concretions incorporate a wide range of other metals, such as Co, Ni, V, Zn, Mo, Al, Cr, Cu, Pb, and Li, as well as REEs (Zhamoida et al. 1996; Baturin 2009; Wasiljeff, Yu, et al. 2025), and are recognised as potential sources for Earth's critical minerals. Moreover, concretions accumulate phosphorus, serving as natural filters for P on the seafloor (Zhamoida et al. 2017). Conversely, during anoxia, P can be released from Fe‐Mn concretions in the water column, thereby forming a temporal source of bioavailable P (Yli‐Hemminki et al. 2016). Fe‐Mn concretions have a notably high growth rate in the Baltic Sea region, estimated at the range of 0.001 to 0.08 mm/year (Majamäki et al. 2025; Zhamoida et al. 1996; Grigoriev et al. 2013; Liebetrau et al. 2002), compared to the rate of 1–5 mm per million years documented for oceanic ferromanganese nodules (e.g., Hein and Koschinsky 2014). The rapid growth of Baltic Sea concretions results from enhanced microbial activity within concretions, which promotes metal capture (e.g., Majamäki et al. 2025; Yli‐Hemminki et al. 2014; Zhang et al. 2002; Sujith et al. 2017). Previous studies based on the 16S rRNA gene sequencing have shown that spheroidal and crust Fe‐Mn concretions in the Baltic Sea are predominantly inhabited by species from phylum Pseudomonadota (previously Proteobacteria), with many Mn‐ and Fe‐oxidisers and reducers, indicating their potential role in regulating the metal cycling within concretions with oxidative and reductive metabolism (Yli‐Hemminki et al. 2014; Reunamo et al. 2017). Furthermore, *Nitrospira* was identified from Baltic Sea concretions, suggesting their potential role in nitrification (Yli‐Hemminki et al. 2014; Reunamo et al. 2017). Communities obtained from the Baltic Sea region resemble those from deep‐sea nodules, sharing also a high abundance of Fe‐ and Mn‐oxidisers and reducers (e.g., Blöthe et al. 2015; Tully and Heidelberg 2013; Molari et al. 2020; Lindh et al. 2017). On the deep‐sea seafloor, microbial communities in nodules differ from those in the surrounding sediments suggesting that nodules serve as specific ecological niches for microbes and there are variations in metabolic potential between nodules and the environment surrounding them (Blöthe et al. 2015; Tully and Heidelberg 2013; Molari et al. 2020).

In our previous study, we conducted a 12‐week pioneering microcosm incubation experiment, which demonstrated that microbial communities enhance metal accumulation in Fe‐Mn concretions (Majamäki et al. 2025). In this study, we conducted a 15‐week microcosm experiment to enable a more detailed analysis of the metal and REE accumulation and, for the first time, to characterise the microbial community composition across three main Fe‐Mn concretion morphotypes in the Gulf of Finland: crust, discoidal, and spheroidal. Our aims were to (1) understand the influence of microbial activity across different Fe‐Mn concretion morphotypes in the incorporation and release of trace metals and rare‐earth elements, using biotic and abiotic treatments, and (2) to characterise the microbial community composition across different concretion morphotypes, using metatranscriptomics for active populations and metagenomics for both active and dormant communities.

Building upon previous work (Majamäki et al. 2025), we hypothesise that microbial activity will enhance metal and REE accumulation in Fe‐Mn concretions, with strongest effect occurring in spheroidal and discoidal morphotypes. We predict that Fe‐Mn concretions are hosted by diverse microbial populations, including active Fe‐ and Mn‐oxidisers that may contribute to metal enrichment. We further hypothesise that microbial activity will be reflected in increased carbon dioxide production, decreased oxygen concentrations, and decreased methane concentrations in microcosms due to methanotrophic activity, which will be supported by microbial community composition data. In contrast, we do not have a clear prediction for nitrous oxide production since both denitrification and nitrification could occur during the experiment.

## 2 Materials and Methods

### 2.1 Study Sites and Collection of Fluffy Mud, Fe‐Mn Concretion, and Sediment Samples

**FIGURE 1:**
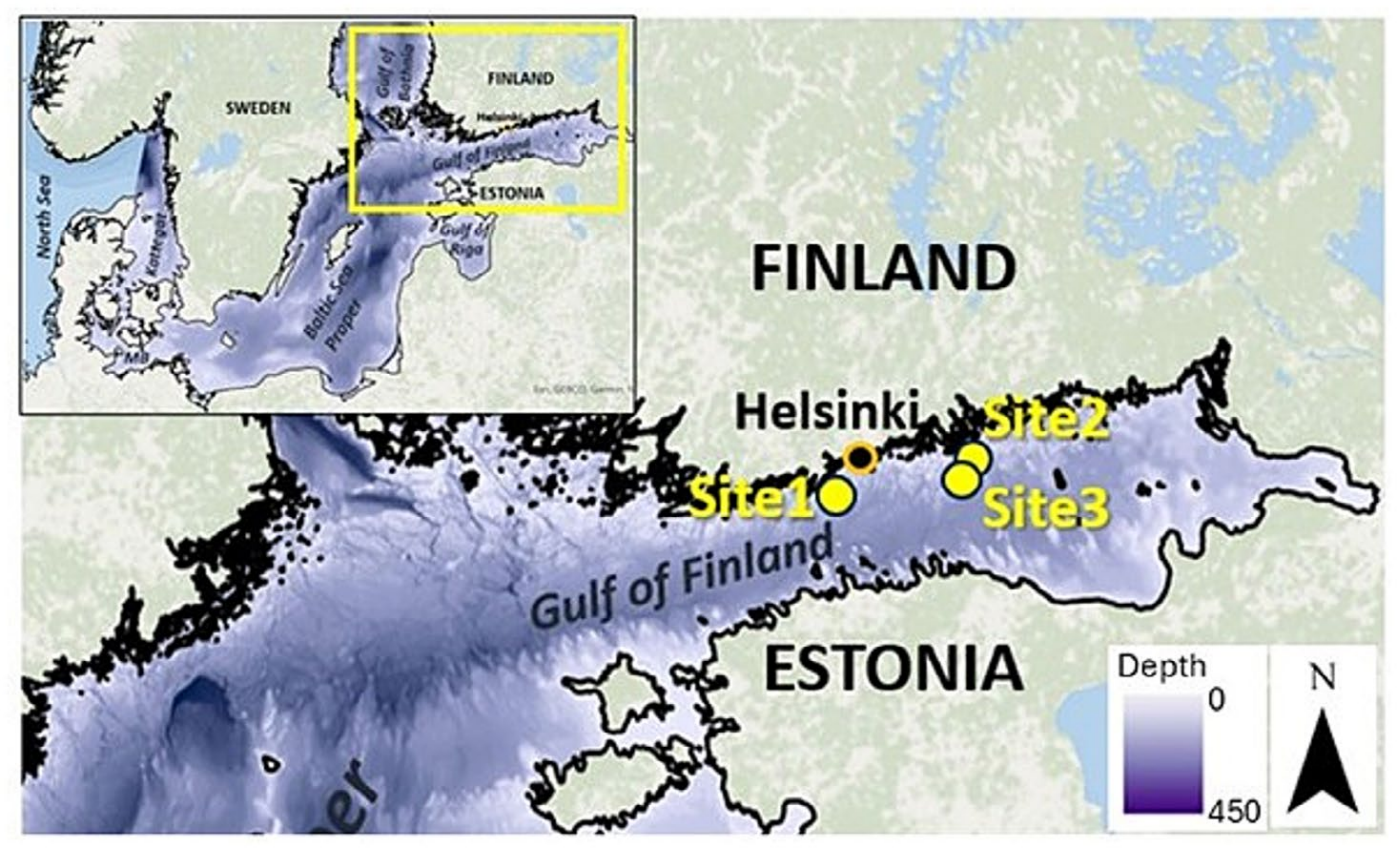
Bathymetric map of the Baltic Sea and Gulf of Finland with the study sites 1, 2, and 3 indicated. MB, Mecklenburg Bight. Base map: ESRI Inc. (Redlands) Ocean Basemap 2018. Baltic Sea bathymetric data: EMODnet Bathymetry 2018 (Thierry et al. 2019).

Sediment core samples from the northern Gulf of Finland, with ferromanganese concretions on the surface, were collected using a box core sampler (20 × 20 cm surface area) during two expeditions in May 2023 (Figure 1 and Table 1). The expeditions revisited the three sites that were previously studied in a microcosm incubation experiment (Majamäki et al. 2025), which were known to contain three main concretion morphologies in the region: crust, discoidal, and spheroidal. Before sampling, the seafloor coverage of the concretions was confirmed with an underwater video camera. Water column profiles of temperature, salinity, and dissolved oxygen (DO) concentration were measured using a Sea & Sun Technology 90 M multiprobe at each sampling site. The pH of seawater was measured on board from water samples collected at 30 m using a Limnos water sampler, the maximum depth accessible with this equipment.

**TABLE 1:** Locations of study sites and the morphotype of ferromanganese concretions collected from the Gulf of Finland.

Site	Sediment core ID	Latitude N (WGS84)	Latitude E (WGS84)	Date	Water depth (m)	Concretion morphotype
1	MGBC‐2023‐1	59°57.941′	24°46.718′	11 May 2023	42	Crust
2	MGBC‐2023‐3	60°05.493′	25°57.789′	22 May 2023	30	Discoidal
3	MGBC‐2023‐2	60°11.483′	26°01.895′	22 May 2023	42	Spheroidal

**FIGURE 2:**
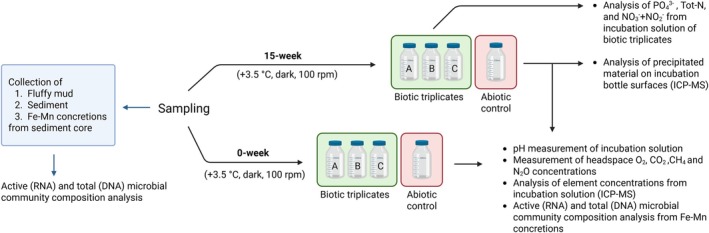
Sampling design and workflow for the microcosm experiment for each Fe‐Mn concretion morphotype: crust, discoidal and spheroidal.

For intrinsic microbial community composition analysis, triplicate samples of surface mud layer (fluffy), Fe‐Mn concretions, and surface sediment were immediately collected into Whirl‐Pak plastic bags (Nasco) from the sediment core and placed on dry ice (Figure 2). The concretions were first rinsed with filtered (pore size 0.22 μm, polyethersulfone membrane) seawater, collected from a depth of 30 m, to remove any excess fluffy mud and sediment. Filters were collected into plastic bags and placed on dry ice. During the 2022 expeditions (Majamäki et al. 2025), triplicate fluffy mud and surface sediment samples, and one Fe‐Mn concretions sample, were collected from each site. The plastic bags containing the samples were kept on dry ice onboard the research vessel and then stored at the −80°C freezer for nucleic acid extraction at the end of each expedition.

Microcosms for incubation experiments were prepared as described in Majamäki et al. (2025). Briefly, 130 mL of autoclaved artificial brackish seawater with a composition similar to water in the Gulf of Finland (salinity 4.7, Yli‐Hemminki et al. 2014; prepared before sampling; Table S1) was added into 250 mL glass bottles. Deviating from Majamäki et al. (2025), the concentrations of Fe and Mn were increased 30‐fold (Table S1) to enhance ferromanganese precipitation. The initial pH of the artificial seawater was measured. All bottles and tools were acid‐washed and autoclaved prior to the experiment. Spoons and tweezers used for sampling were cleaned with 70% ethanol between samples. The crust concretions were broken down into smaller pieces to fit into the bottles, whereas three to four individual discoidal and spheroidal concretions were placed into each bottle. The glass bottles containing the concretions were stored in the dark at +8°C onboard the r/v Geomari and then transported directly to the GTK laboratory in Espoo at the end of each expedition. Bottles with Fe‐Mn concretions were weighed and then placed in sample storage at +3.5°C for the start of incubation experiments.

### 2.2 Incubation Experiments

The microcosm incubation experiments were conducted as described in Majamäki et al. (2025). Briefly, each incubation experiment was conducted using bottles capped with rubber septa: three biotic microcosm bottles (biotic triplicates) and one abiotic control (Figure 2). Parallel incubation experiments were performed for each concretion morphotype. Deviating from Majamäki et al. (2025), a final concentration of 50 mM sodium azide (NaN_3_) was added to the abiotic controls (15 mM was used in Majamäki et al. 2025) to eliminate microbial activity (Cabrol et al. 2017; Sujith et al. 2017), and the incubation time was extended to 15 weeks (12 weeks in Majamäki et al. 2025). To simulate seafloor conditions, all microcosms were incubated in the dark at +3.5°C in an orbital shaker at 100 rpm. Biotic triplicates and abiotic control were sacrificed 1 day after sampling (0‐week) and after 15 weeks of incubation (15‐week). Headspace gas samples were collected in duplicates into pre‐evacuated 8 mL Exetainer tubes (Labco, UK) through rubber septa using a needle and syringe, and concentrations of carbon dioxide (CO_2_), methane (CH_4_), nitrous oxide (N_2_O), and oxygen (O_2_) were measured as described in Section 2.3. Concentrations of dissolved phosphorus, metals, and rare earth elements (REEs) in the incubation solutions were analysed as described in Section 2.3. These elements were also analysed from the material precipitated on the inner walls of the bottles in 15‐week microcosms, described in Section 2.3. Concretions were collected from microcosms into Whirl‐Pak plastic bags using flame‐sterilised and cooled tweezers, flash‐frozen on dry ice, and stored in the −80°C for nucleic acid extraction (Section 2.4). Concretions were collected and stored similarly from bottles in the microcosm experiment in 2022 for nucleic acid extraction (Majamäki et al. 2025). The pH of the incubation solution was measured. From the 15‐week biotic triplicates, phosphate (PO_4_^3−^), total nitrogen (Tot‐N), and nitrate and nitrite (NO_3_^−^ + NO_2_^−^) concentrations were analysed from the incubation solution as described in Section 2.3.

### 2.3 Gas Concentration Measurements and Geochemical Analysis

Headspace gas concentrations were measured using a custom gas chromatography system Agilent 7890B (Soinne et al. 2021). Sample separation was conducted using a combination of three Hayesep Q 80/100 (Unimetal) and one HP‐Molsieve Plot column (Soinne et al. 2021). Gases were measured using thermal conductivity (O_2_, CO_2_), flame ionisation (CH_4_), or electron capture (N_2_O) detectors. Detection limits were 50 ppm for CO_2_, 0.2 ppm for CH_4_, 0.03 ppm for N_2_O, and 20 ppm for O_2_.

To assess the metal concentrations in the incubation solution, 100 mL of solution from each bottle was filtered through a 0.2 μm cellulose acetate filter and stored at +3.5°C in plastic bottles containing a final concentration of 4.5% (v/v) nitric acid (HNO_3_) until analysis. Additionally, 100 mL of artificial seawater (as described above) was stored in a plastic bottle to obtain the initial composition of the incubation solution. To assess the metal concentrations that were precipitated on the inner walls of incubation bottles, the 15‐week microcosms were emptied entirely and rinsed several times with deionised water to remove excess Fe‐Mn concretion residues. Five millilitres of 10% HNO_3_ was added to the bottles, and the acid was heated at +60°C for 24 h to release any precipitated material from the bottle walls. The next day, acid was collected into test tubes, and the bottles were rinsed with 5 mL of MQ water three times. The rinsed water was added to the test tube containing the acid, resulting in a 10 mL solution with a final concentration of 5% (v/v) HNO_3_. The test tubes were stored at +3.5°C until analysis. Solutions were measured at the Hellabs laboratory of University of Helsinki, Finland, using an Agilent 8900 triple quadrupole ICP‐MS (ICP‐QQQ) system to assess the trace element (non‐REE) concentrations of P, Mn, Fe Co, Ni, V, Zn, Mo, Al, Cr, Cu, Pb, and Li, and REE concentrations of La, Ce, Pr, Nd, Sm, Eu, Gd, Tb, Dy, Y, Ho, Er, Yb and Lu. The original trace element concentrations (Table S4) were normalised to the measured concentrations of artificial seawater (Figure 5, lower panels; Tables S2 and S5). The concentration differences were calculated as relative changes by subtracting the 0‐week values from the 15‐week values and then dividing by the 0‐week value (Figure 5, upper panels; Table S6). Original rare earth elements and yttrium (REY) concentrations (Tables S8 and S10) were normalised to Post‐Archean Australian Shale (PAAS) values (Figure 7; Tables S9 and S11) (Pourmand et al. 2012). Detection limits for trace elements were (ppb): Al 0.25, P 0.06, V 0.01, Cr 0.02, Mn 0.01, Fe 0.08 Co 0.01, Ni 0.02, Cu 0.03, Zn 0.03, Mo 0.03, Pb 0.01, and Li 0.50. Detection limits for REEs were (ppb): Y 0.113, La 0.001, Ce N/A, Pr N/A, Nd 0.007, Sm 0.002, Eu 0.004, Gd 0.005, Tb 0.004, Dy 0.002, Ho 0.019, Er 0.002, Yb 0.005, and Lu 0.008.

To assess PO_4_^3−^, Tot‐N, and NO_3_^−^ + NO_2_^−^ concentrations, 100 mL of incubation solution from each of the 15‐week biotic triplicates was filtered through a 0.2 μm cellulose acetate filter and frozen at −20°C until analysis. Analysis of the abiotic controls was not performed because the addition of NaN_3_ influenced the Tot‐N and NO_3_ + NO_2_ results. Solutions were measured at Tvärminne Zoological Station, University of Helsinki, with a Thermo Scientific Aquakem 250 analyser using Grasshoff's (1976) and Koroleff's (1979) methods, modified for an automatic analyser (Koistinen et al. 2020a, 2020b). The PO_4_^3−^ concentration was analysed using the traditional antimony‐molybdate method (Koistinen et al. 2020a). Tot‐N was analysed through alkaline K_2_S_2_O_8_ oxidation to oxidise inorganic and organic nitrogen compounds to nitrate, and NO_3_ + NO_2_ concentrations were measured by reducing nitrate to nitrite with vanadium chloride, and nitrite was determined using an azo dye (Koistinen et al. 2020b). Detection limits were 20 μg/L for Tot‐N, 1 μg/L for NO_3_ + NO_2_, and 1 μg/L for PO_4_^3−^.

### 2.4 Nucleic Acid Extraction and Purification

Approximately 1 g from each concretion and sediment sample was weighed in duplicate, while duplicates of 0.5 g were weighed from each fluffy sample. Samples were transferred into 7 mL extraction tubes (Omni International) on dry ice, using flame‐sterilised and cooled‐down tweezers. Beads from two Lysing Matrix E tubes (MP Biomedicals) were added to each tube. Nucleic acids were extracted using the modified CTAB, phenol‐chloroform, and bead‐beating protocol (Griffiths et al. 2000; DeAngelis et al. 2009; Viitamäki et al. 2022). The protocol was further modified by adding a sodium pyrophosphate buffer in the cell lysis step to enhance nucleic acid desorption from (hydr)oxides, optimising the protocol for samples with high metal (hydro)oxide content and low nucleic acid content (Kallmeyer et al. 2008; Zhang et al. 2008; Muto et al. 2017).

On ice, 1 mL of sodium pyrophosphate decahydrate (Na_4_P_2_O_7_ · 10H_2_O: 0.2 M for concretions and 0.1 M for sediments, fluffy and filters), 1 mL of CTAB buffer (10% CTAB in 1 M NaCl and 0.5 M phosphate buffer in 1 M NaCl), and 100 μL 0.1 M ammonium aluminium sulfate (NH_4_(SO_4_)2 · 12 H_2_O) were added into the extraction tubes and vortexed. Then, 1 mL of phenol:chloroform:isoamyl alcohol (25:24:1) was added, and the tubes were bead‐beaten with Omni Bead Ruptor 24 with Cryo Cooling Unit (OMNI International) at +4°C, 5.5 m s^−1^ for 30 s, followed by centrifugation for 5 min. In Fe‐Mn concretion samples, a thin, brownish film remained above the aqueous layer, and they were therefore re‐extracted with phenol by transferring the aqueous phase into a new Eppendorf tube with 1 volume of phenol:chloroform: isoamyl alcohol (25:24:1), vortexed, and centrifuged for 5 min. The aqueous phase was transferred to new Eppendorf tubes with 1 volume of chloroform, vortexed, and centrifuged for 5 min. Nucleic acids were precipitated with 2 volumes of PEG6000 (30% in 1.6 M NaCl) for 1–2 h at room temperature and centrifuged for 10 min. Pellets containing nucleic acids were washed with cold 70% ethanol and centrifuged for 5 min. Each sample was extracted twice by adding 1 mL CTAB and 0.5 mL sodium pyrophosphate again to the bead‐beating tube to maximise nucleic acid yields. Pellets were dissolved in 25 μL of Buffer EB and 250 μL Buffer RLT (Qiagen) with 1% β‐mercaptoethanol. All centrifugations were done at +4°C and 16,000 g. All steps were performed using nuclease‐free labware, reagents were treated with 0.1% diethylpyrocarbonate (DEPC), and equipment and surfaces were cleaned with RNase AWAY (Thermo Scientific) to avoid nucleic acid and RNase contamination.

The DNA and RNA were purified using the AllPrep DNA/RNA Mini Kit with DNase I treatment (Qiagen). Purified DNA was stored at −20°C, and RNA at −80°C. RNA concentrations and RNA integrity number (RIN) were measured using the Bioanalyzer 2100 Nano/Pico Chip with Prokaryote Total RNA Assay (Agilent). PCR with universal 16S rRNA gene primers (pA) forward [5′‐AGAGTTTGATCCTGGCTCAG‐3′] and (pH) reverse [5′‐AAGGAGGTGATCCAGCCGCA‐3′] (Edwards et al. 1989) with 20 cycles was conducted to confirm that the RNA was DNA‐free. Fluffy samples observed to contain DNA contamination were DNase‐treated a second time using the AllPrep DNA/RNA Mini Kit with DNase I treatment and then rechecked by PCR.

### 2.5 Library Preparation, Sequencing and Bioinformatics

DNA from each fluffy, sediment, and Fe‐Mn concretion sample collected immediately after sampling (intrinsic samples) was pooled. Concentration and quality control measures were performed at Novogene GmbH (Munich, Germany) prior to metagenomic DNA library preparation. Shotgun metagenomic sequencing was performed using the Illumina NovaSeq X Plus platform with a paired‐end, 150‐bp read length strategy at Novogene GmbH.

For RNA samples, duplicates from each extracted sample were pooled, and complementary DNA (cDNA) libraries were prepared using the NEBNext Ultra II Directional RNA Library kit for Illumina and NEBNext Multiplex Oligos for Illumina (96 Unique Dual Index Primer Pairs) (New England Biolabs). Library concentrations were measured using a Qubit 4 Fluorometer with the 1X dsDNA High Sensitive/Broad Range Assay Kit (Invitrogen), and library size and quality were assessed by gel electrophoresis. Libraries with low concentrations (< 1 μg/μL) and those containing small cDNA fragments (~80 bp for primers or ~128 bp for adaptor‐dimer) were disqualified and not included in the pooled library for sequencing. The pooled library was sequenced using the Illumina NovaSeq X Plus platform with a paired‐end, 150‐bp read length strategy at Novogene GmbH.

Metagenomic raw sequence data was trimmed at Novogene GmbH by removing reads containing adapters, reads with *N* > 10%, and reads in which more than 50% of the total bases contained low‐quality bases (Qscore ≤ 5). Metatranscriptomic raw sequence data was trimmed from adapters, short reads, and low‐quality reads using Cutadapt v. 4.9 (Martin 2011), with a minimum read length set to 50 bp and the nextseq‐trim option set to 20. Sequence quality was assessed using FastQC v. 0.11.9 (Andrews 2010) and MultiQC v. 1.19 (Ewels et al. 2016) before and after trimming.

For trimmed metagenomic and metatranscriptomic data, Phyloflash v. 3.4.2 (Gruber‐Vodicka et al. 2020), with the SILVA SSURef NR99 database release 138.2 (Quast et al. 2013), was used for taxonomic classification of SSU rRNA sequences and for identification of microbial community composition. An error in SILVA release 138.2 was fixed by changing the incorrect genus name, *Candidatus Nitrosoarchaeum*, to *Incertae Sedis* in the SAR324 clade (Marine group B) bacterial phylum.

### 2.6 Statistical Analysis

Data visualisation and statistical calculations were done using R (R Core Team 2025, version 4.5.1) in RStudio, using the following packages: tidyr (v. 1.3.1, Wickham et al. 2024), dplyr (v. 1.1.4, Wickham et al. 2023), ggplot2 (v. 4.0.0, Wickham 2016), phyloseq (v. 1.46.0, McMurdie and Holmes 2013), RColorBrewer (v. 1.1‐3, Neuwirth 2022), car (v. 3.1.5, Fox and Weisberg 2019), rstatix (v. 0.7.3, Kassambara 2025), forcats v. 1.0.1 (Wickham 2025), vegan v. 2.1‐1 (Oksanen et al. 2025), ggrepel v. 0.6.6 (Slowikowski 2024), and cowplot v. 1.2.0 (Wilke 2025). For stacked barplots (Figures 9 and 11), raw sequence counts were converted to relative abundances by dividing the count of each taxon by the total counts per sample. Phylum‐level counts were summed across all samples, and the 15 phyla with the highest total counts were selected for visualisation. For the heatmap (Figure 12), raw sequence counts were first converted to relative abundances and further square‐root transformed (Hellinger transformation). Counts were summed across all samples, and the 70 most abundant genera were selected for visualisation. Eukaryota were included in the domain‐level visualisation (Figure 9, upper panel) but were excluded from the phylum‐level analysis (Figure 9, lower panel; Figure 11) and the heatmap visualisation (Figure 12) to keep focus on archaea and bacteria that are expected to contribute most to metal‐cycling processes. Genera with uncertain classification (Incertae Sedis) were changed to NA prior to plotting the heatmap.

Principal coordinate analysis (PCoA) was performed using Bray–Curtis distances calculated from relative abundances of raw sequence counts (Figures 10 and 13). Differences in community composition across sites/morphotypes (Figure 13; crust, discoidal, and spheroidal) and environments (Figure 10; Fe‐Mn concretions, fluffy mud, sediment) were tested using permutational multivariate analysis of variance (PERMANOVA) based on Bray–Curtis distances with the adonis2 function in vegan (Oksanen et al. 2025). Eukaryota were included in the PCoA of community composition across environments (Figure 10) but were excluded from the PCoA across different sites/morphotypes (Figure 13) to keep focus on archaea and bacteria that are expected to contribute most to metal‐cycling processes. Pairwise comparisons between sites and environments were performed, and the proportion of variance explained by each factor was quantified using the PERMANOVA *R* ^2^ statistic, and statistical significance was assessed using permutation‐based (999) *p* values. *p* values were adjusted using the Benjamini–Hochberg (BH) method. The threshold for statistical significance was defined as adjusted *p* ≤ 0.05. Sample sizes among each tested site/morphotype were *n* = 6, except for the 2022 site 3 (spheroidal) RNA‐derived samples, where *n* = 5. Sample sizes among each tested environment were: in 2023, fluffy (*n* = 12), sediment (*n* = 7), and concretion (*n* = 12), and in 2022, fluffy (*n* = 12), sediment (*n* = 3), and concretion (*n* = 6). Homogeneity of multivariate dispersion was assessed using the betadisper function in vegan (Oksanen et al. 2025) and significance using permutation testing.

Arrows representing elemental vectors were fitted onto the PCoA ordinations using the envfit function in vegan (Oksanen et al. 2025) to visualise correlations between elemental concentrations and community composition (Figure 13). Element concentrations were first normalised to measured values of artificial seawater (Table S2) and then log_10_‐transformed to reduce skewness. The strength of each correlation (*R* ^2^) and its statistical significance were assessed using permutation tests included in envfit (999 permutations), with *p* values adjusted using the BH method. The threshold for statistical significance was defined as adjusted *p* ≤ 0.05.

Shannon diversity (*H*′) was calculated for each sample using the estimate_richness function in phyloseq (McMurdie and Holmes 2013). Differences in Shannon diversity between sites/morphotypes (crust, discoidal, and spheroidal) and environments (Fe‐Mn concretions, fluffy mud, and sediment) were tested using one‐way analysis of variance (ANOVA) with Tukey's HSD post hoc test. Normality of residuals was assessed using the Shapiro–Wilk test, and homogeneity of variances was assessed using Levene's test in the car package (Fox and Weisberg 2019). Levene's test was violated for the ANOVA results in Fe‐Mn concretion samples collected from the incubation experiment in 2022, and therefore, Welch's ANOVA followed by Games–Howell post hoc test in the rstatix package (Kassambara 2025) was used instead. Sample sizes among each tested site/morphotype were *n* = 12, except for the 2022 site 3, where *n* = 11. Sample sizes among each tested environment were: in 2023, fluffy (*n* = 12), sediment (*n* = 7), and concretion (*n* = 12), and in 2022 fluffy (*n* = 12), sediment (*n* = 3), and concretion (*n* = 6). Results are presented as mean ± SD. The threshold for statistical significance was defined as adjusted *p* ≤ 0.05.

## 3 Results

### 3.1 Water‐Column Profiles and Occurrence of Fe‐Mn Concretions at Sampling Sites

**FIGURE 3:**
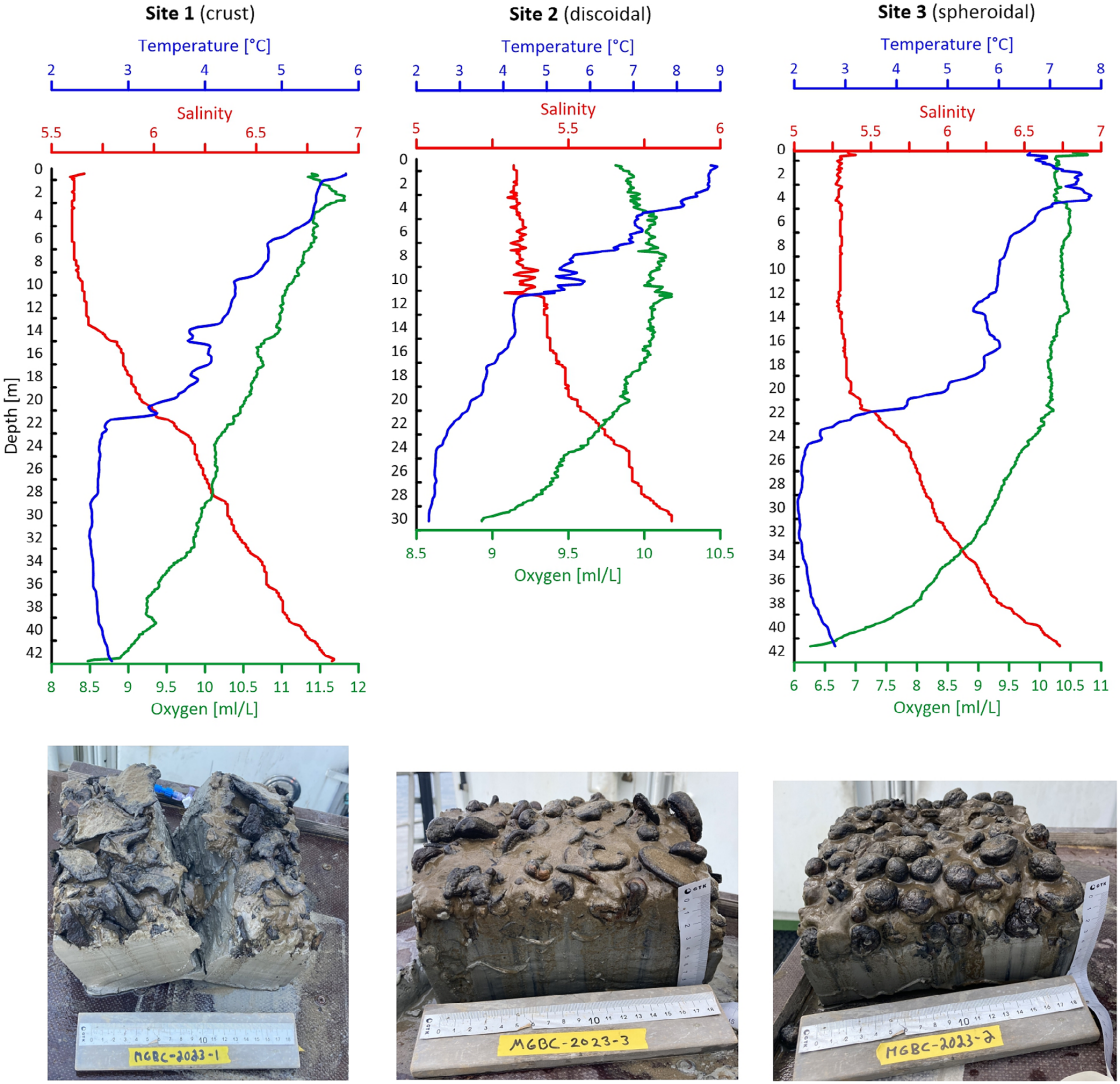
Vertical water‐column profiles of temperature, DO concentration, and salinity and photos of sediment cores with ferromanganese concretions on their surface from three study sites in the Gulf of Finland. Site 1 was characterised by crust concretions, site 2 by discoidal concretions, and site 3 by discoidal and spheroidal concretions. Only spheroidal concretions were collected from site 3.

At all three sampling sites, seawater temperature and dissolved oxygen (DO) concentration decreased with depth, while salinity increased (Figure 3). At site 1, the bottom temperature was +2.8°C, and DO was 8.5 mL/L. The salinity at the bottom was 6.9. At site 2, the bottom temperature was +2.3°C, with a DO of 8.9 mL/L and salinity of 5.8. At site 3, the bottom temperature was +2.8°C, DO was 6.3 mL/L, and salinity was 6.7. The seawater pH values at the 30 m depth were 7.5, 7.2, and 7.2 at sites 1, 2, and 3, respectively.

Fe‐Mn concretions were present on the surface of each sediment core (Figure 3). At site 1, the concretions had a crust‐type morphology, whereas at site 2, they were discoidal. At site 3, both discoidal and spheroidal concretions were present. Brownish, organic‐rich ‘fluffy’ mud covered the concretions. The sediment underlying the concretions was grey glaciolacustrine rhythmite silt and clay at site 1. At site 2, there was a 3‐cm layer of Fe‐monosulphide darkened fine sand beneath the concretions, which was underlain by grey glaciolacustrine rhythmite silt and clay. At site 3, the underlying sediment was Fe‐monosulphide banded grey postglacial lacustrine silty clay (sediment identification follows Virtasalo et al. 2007, 2014).

### 3.2 Microcosm Solution pH and the Headspace Gas Concentrations

The mean weight for site 1 (crust) Fe‐Mn concretions was 17.4 g (±2.6) in 0‐week bottles, and 17.9 g (±1.3) in 15‐week bottles. For site 2 (discoidal), the mean weight was 24.0 g (±1.1) in 0‐week and 17.8 g (±0.9) in 15‐week. For site 3 (spheroidal), the mean weight was 26.8 g (±2.3) in 0‐week and 25.2 g (±2.6) in 15‐week.

The artificial brackish seawater (Yli‐Hemminki et al. 2014; Table S1) had an initial pH of 7.0. In microcosms, the mean pH of the incubation solution ranged from 6.3 to 7.1 in biotic triplicates and from 6.9 to 7.3 in abiotic controls during 15‐week of incubation (Table 2). In site 1 microcosms, both in the biotic triplicates and the abiotic control, the mean pH remained constant before and after 15 weeks of incubation. In site 2, the mean pH increased by 0.6 units in the biotic triplicates and by 0.2 units in the abiotic control. In site 3 microcosms, the mean pH increased by 0.8 units in the biotic triplicates and by 0.2 units in the abiotic control.

**TABLE 2:** The pH values of the incubation solutions from biotic triplicates and abiotic controls at the beginning (0 week) and the end (15 weeks) of the experiment.

Biotic triplicates	Site 1 (crust)		Site 2 (discoidal)		Site 3 (spheroidal)	
	0‐week	15‐week	0‐week	15‐week	0‐week	15‐week
A	6.6	6.9	6.5	7.2	6.2	7.2
B	6.7	6.8	6.4	6.9	6.3	7.1
C	7.1	6.7	6.4	6.8	6.3	6.9
Mean (std)	6.8 (±0.2)	6.8 (±0.1)	6.4 (±0.0)	7.0 (±0.2)	6.3 (±0.1)	7.1 (±0.1)
Abiotic control	7.1	7.1	7.1	7.3	6.9	7.1

**FIGURE 4:**
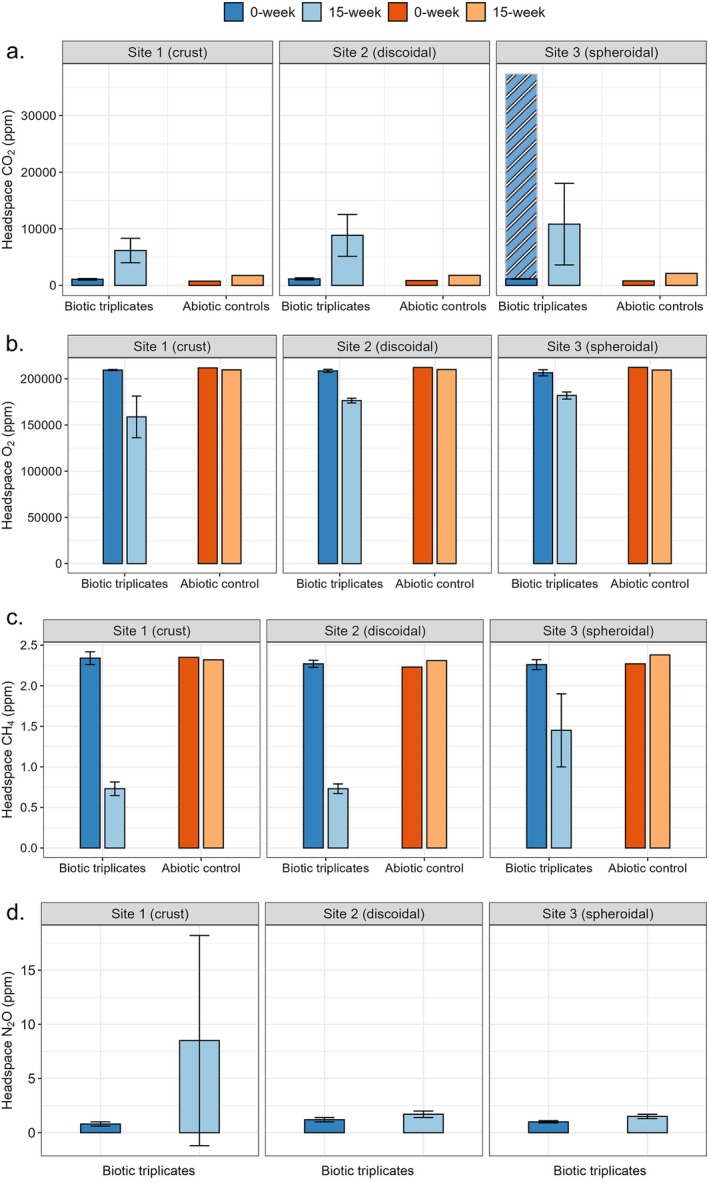
Mean headspace (a) CO_2_, (b) O_2_, and (c) CH_4_ concentrations in biotic triplicates (*n* = 3, except for CO_2_ site 3 0‐week, where *n* = 2) and abiotic control (*n* = 1) at the start (0‐week) and at the end (15‐week) of the experiments. (d) Mean N_2_O headspace concentrations are presented only for biotic triplicates (*n* = 3) at 0‐week and 15‐week. Error bars show the standard deviation of the biotic triplicates.

Headspace CO_2_ concentrations increased strongly in biotic triplicates from all sites during 15 weeks of incubation (Figure 4a and Table S3). In abiotic controls, a much smaller increase was observed. In site 1, CO_2_ concentration increased the most, being approximately 5.8 times higher in the 15‐week microcosm (6168 ± 2153 ppm) than in the 0‐week microcosm (1073 ± 135 ppm). In the respective abiotic control, the increase was 2.3 times higher (1750 ppm in the 15‐week microcosm; 746 ppm in the 0‐week microcosm). In site 2 biotic triplicates, CO_2_ concentration was 7.7 times higher in the 15‐week microcosm (8839 ± 3696 ppm) than in the 0‐week microcosm (1147 ± 185 ppm). In the respective abiotic control, the concentration increased 2.1 times over 15 weeks, from 853 ppm to 1776 ppm. In site 3 biotic triplicates, concentration increased 9.4 times in the 15‐week microcosm (10,821 ± 7207 ppm) compared to the 0‐week microcosm (1153 ± 71 ppm). In one biotic replicate, exceptionally high CO_2_ production was observed in the headspace of the 0‐week bottle (37,328 ppm), and this result is illustrated as a dashed bar in Figure 4a. This outlier replicate was excluded from the CO_2_ analyses. In abiotic control, the concentration was 2.7 times higher after 15 weeks (2120 ppm) than at 0 weeks (790 ppm).

Headspace O_2_ concentrations decreased in biotic triplicates but remained nearly unchanged in abiotic controls in all sites (Figure 4b and Table S3). In site 1 biotic triplicates, the mean O_2_ concentration decreased 1.3 times in the 15‐week microcosm (158,788 ± 22,552 ppm) compared to the 0‐week microcosm (209,469 ± 529 ppm). In abiotic control, O_2_ concentration remained almost unchanged, decreasing from 211,847 ppm (0‐week) to 209,683 ppm (15‐week). In site 2 biotic triplicates, the concentration was 1.2 times lower in the 15‐week microcosm (176,382 ± 2590 ppm) than in the 0‐week microcosm (208,628 ± 1596 ppm). In abiotic control, concentration decreased from 212,246 to 209,999 ppm. In site 3 biotic triplicates, O_2_ concentration decreased 1.1 times, from 206,537 ± 3292 ppm (0‐week) to 181,914 ± 3834 ppm (15‐week). In abiotic controls, concentration decreased from 212,329 to 209,540 ppm.

Headspace CH_4_ concentrations decreased in biotic triplicates in all sites during 15 weeks of incubation (Figure 4c and Table S3), with the largest decrease observed at sites 1 and 2. In abiotic controls, changes in CH_4_ concentrations were not observed because they were below the detection limit. In site 1 biotic triplicates, the mean CH_4_ concentration was approximately 3.2 times lower in the 15‐week microcosm (0.73 ± 0.08 ppm) compared to the 0‐week microcosm (2.34 ± 0.08 ppm). In site 2 biotic triplicates, the concentration was 3.1 times lower in the 15‐week microcosm (0.73 ± 0.06 ppm) than in the respective 0‐week microcosm (2.27 ± 0.04 ppm). In site 3 biotic triplicates, the CH_4_ concentration decreased 1.6 times, from 2.3 ± 0.06 ppm (0 week) to 1.5 ± 0.45 ppm (15 weeks).

The headspace N_2_O concentrations increased in biotic triplicates from all sites after 15 weeks of incubation (Figure 4d and Table S3). The increase was most substantial in site 1 biotic triplicates, where mean N_2_O concentration increased 10.2 times in the 15‐week microcosm (8.47 ± 9.69 ppm) compared to the 0‐week microcosm (0.83 ± 0.18 ppm). In site 2, the mean N_2_O concentration increased 1.4 times, from 1.21 ± 0.16 ppm (0 week) to 1.74 ± 0.28 ppm (15 weeks), and in site 3, the concentration increased 1.5 times from 0.99 ± 0.07 ppm (0 week) to 1.46 ± 0.17 ppm (15 weeks). Sodium azide (NaN_3_), which was used in abiotic controls, resulted in excessive N_2_O formation in the headspace (Hendrix et al. 2019), and results were thus irrelevant and excluded from Figure 4d. In site 1, abiotic controls, N_2_O concentration increased from 5.7 to 51.3 ppm; in site 2, the concentration increased from 14.7 to 50.1 ppm; and in site 3, the increase was from 8.4 to 50.6 ppm.

### 3.3 Trace Metal and Rare Earth Element Concentrations in Microcosms

**FIGURE 5:**
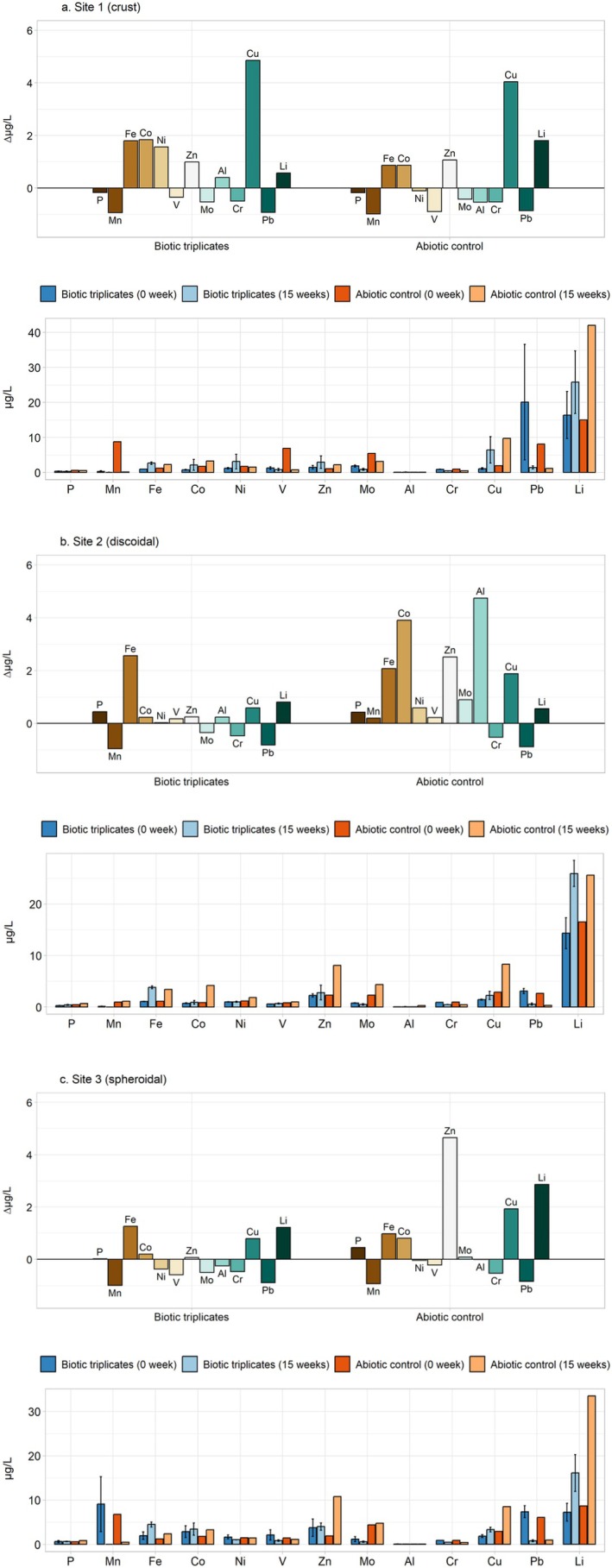
Mean concentrations of P, Mn, Fe Co, Ni, V, Zn, Mo, Al, Cr, Cu, Pb, and Li in the solutions of biotic triplicates (*n* = 3) and abiotic control (*n* = 1) at the beginning (0 week) and at the end (15 weeks) of the incubation (lower panels; Table S5), and the concentration differences between the 0 week and 15 weeks incubation (upper panels; Table S6) in (a) site 1 (crust), (b) site 2 (discoidal) and (c) site 3 (spheroidal). In the upper panels, bars with positive values have labels above them, while bars with negative values have labels below them. The concentrations are normalised with the measured values of artificial seawater (Table S2). The concentration differences are expressed as the relative change, calculated by subtracting the 0‐week values from the 15‐week values and then dividing by the 0‐week value. Error bars show the standard deviation of the biotic triplicates.

In site 1 (crust), element concentrations in the microcosm solution were generally higher in biotic triplicates compared to abiotic control after 15 weeks of incubation (Figure 5a). Only concentrations of Zn, Mo, Pb, and Li were lower in biotic triplicates than in abiotic control. In biotic triplicates, Fe Co, Ni, Zn, Al, Cu, and Li concentrations increased, with Cu showing the strongest increase after the incubation period. Concentrations of P, Mn, V, Mo, Cr, and Pb decreased in biotic triplicates. In abiotic controls, Fe Co, Zn, Cu, and Li concentrations increased in solution after incubation, while P, Mn, Ni, V, Mo, Al, Cr, and Pb decreased.

In site 2 (discoidal), most element concentrations (Al, V, Mn Co, Ni, Cu, Zn, Mo) were lower in the solution of biotic triplicates compared to the abiotic control after a 15‐week incubation (Figure 5b). In the biotic triplicates, concentrations of P Co, Ni, V, Zn, Al, Cu, and Li increased slightly, whereas Fe increased the strongest. In contrast, Mn, Mo, Cr, and Pb concentrations decreased in the biotic triplicates. In the abiotic control, all concentrations except Cr and Pb increased. Concentrations of P, Mn, Ni, V, Mo, and Li showed a slight increase, whereas Fe Co, Zn, Al, and Cu increased noticeably.

In site 3 (spheroidal), most element concentrations (Al, P, V, Mn Co, Ni, Cu, Zn, Mo, Pb, Li) were lower in the solution of biotic triplicates compared to abiotic control after incubation (Figure 5c). In biotic triplicates, P, Fe Co, Zn, Cu, and Li concentrations showed a slight increase, while Mn, Ni, V, Mo, Al, Cr, and Pb decreased. In the respective abiotic control, concentrations of P, Fe Co, Zn, Mo, Cu, and Li increased, with Zn, Cu, and Li showing the greatest increases. The Mn, Ni, V, Al, Cr, and Pb decreased slightly in abiotic control.

It should be noted that only one abiotic treatment and three biotic treatments were used in incubation experiments, which limits the strength of statistical comparisons between these treatments. Furthermore, there was high variability in biotic triplicates, and the error bars of standard deviation are thus not presented in the figures showing the difference between 0‐week and 15‐week incubation (Figure 5, upper panels; Table S6).

**FIGURE 6:**
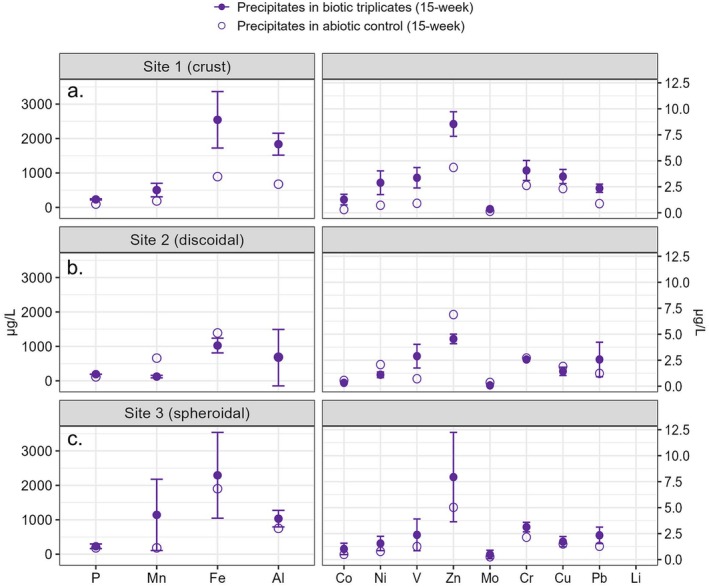
Mean concentrations of P, Mn, Fe, Al Co, Ni, V, Zn, Mo, Cr, Cu, and Pb in precipitates recovered by acid washing from the walls of 15‐week biotic (*n* = 3) and abiotic (*n* = 1) microcosms bottles from (a) site 1 (crust), (b) site 2 (discoidal), and (c) site 3 (spheroidal). Li concentrations were below the detection limit. Error bars show the standard deviation in biotic triplicates.

In all three morphotypes, the precipitated material recovered by acid‐washing from the inner walls of the 15‐week incubation bottles showed elevated concentrations in both biotic triplicates and abiotic controls (Figure 6 and Table S7). In sites 1 (crust) and 3 (spheroidal), concentrations of P, Mn, Fe, Al Co, Ni, V, Zn, Mo, Cr, Cu, and Pb were higher in the biotic triplicates than in the corresponding abiotic controls. In site 2 (discoidal), only concentrations of P, V, and Pb were higher in the biotic triplicates compared to the abiotic control, whereas the concentrations of other metals were more elevated in the abiotic control. The highest concentrations were observed for P, Mn, Fe, and Al across all sites, while the concentrations of the other elements (Co, Ni, V, Zn, Mo, Cr, Cu, Pb) remained comparatively lower.

**FIGURE 7:**
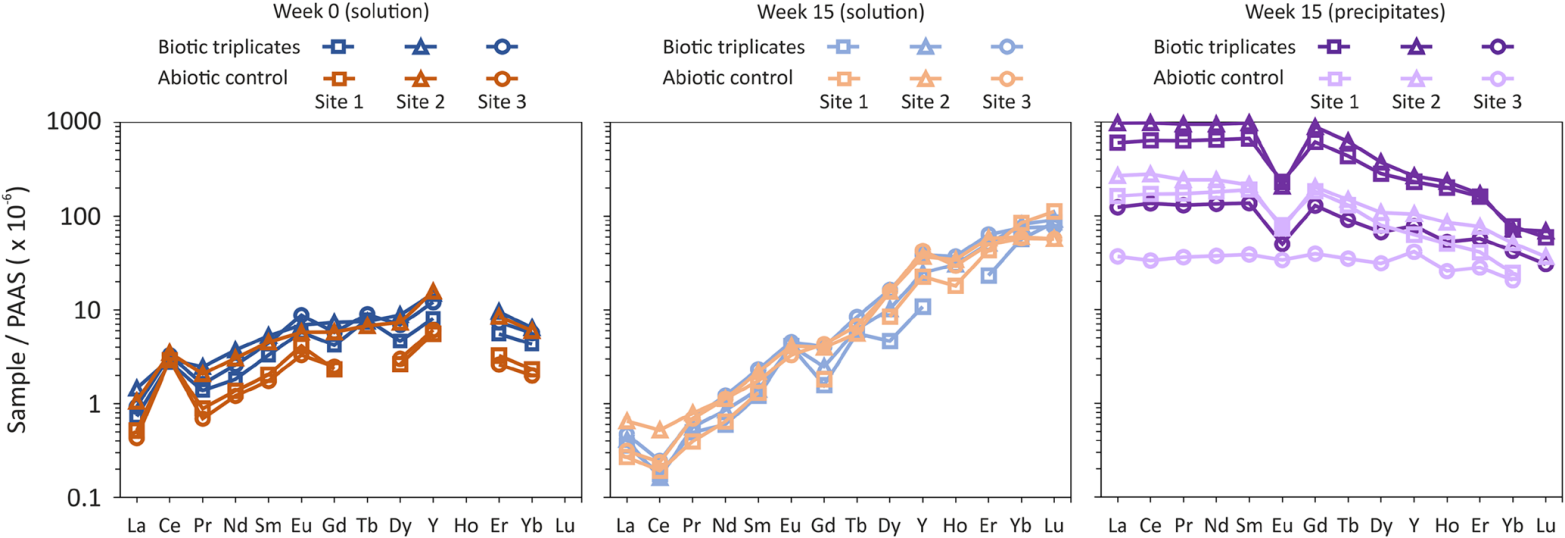
The post‐Archean Australian Shale (PAAS) normalised REY patterns of the solutions at 0‐ and 15‐week and in precipitates recovered by acid washing from 15‐week biotic (*n* = 3) and abiotic (*n* = 1) microcosm bottles.

REY concentrations showed variability across sites (1–3) during the 15‐week experiment (Figure 7 and Table S9). At week 0 in the solution, site 2 showed the highest REY concentration in both the biotic triplicates and abiotic control, followed by site 3 and site 1. After the 15‐week incubations, the sum of REY (ΣREY) increased in the solution of all sites except at site 1 biotic triplicates, where the concentrations decreased slightly. More specifically, the sum of light REE (ΣLREE; La–Nd) decreased and the sum of heavy REE (ΣHREE; Ho–Lu) increased in all samples and treatments, whereas the sum of middle REE (ΣMREE; Sm–Dy) showed differing behaviours. In sites 1 and 2, the ΣMREE concentration decreased in the biotic triplicates but increased in the abiotic controls. In site 3, ΣMREE increased in both treatments. However, these trends were affected largely by increases in Dy, while most other MREEs (Sm–Tb) showed decreasing concentrations during the experiment. The main exception was the site 3 abiotic control, where both Gd and Dy increased while the remaining MREEs were generally unchanged.

In the precipitated material, recovered by acid‐washing from the bottle walls at the end of the experiment (week 15 precipitates), biotic triplicates exhibited higher REY concentrations compared to respective abiotic controls in all samples (Figure 7 and Table S11). Specifically, LREE concentrations were higher in the biotic triplicates. All biotic samples and abiotic controls of sites 1 (crust) and 2 (discoidal) showed a negative Eu anomaly. In site 3 (spheroidal), no Eu anomaly was observed. No Cerium (Ce) anomaly was observed in any of the sampled precipitates.

### 3.4 Tot‐N and NO_2_ ^−^ + NO_3_ ^−^ and PO_4_^3^ ^−^ Concentrations in Artificial Seawater and 15‐Week Biotic Triplicates

**FIGURE 8:**
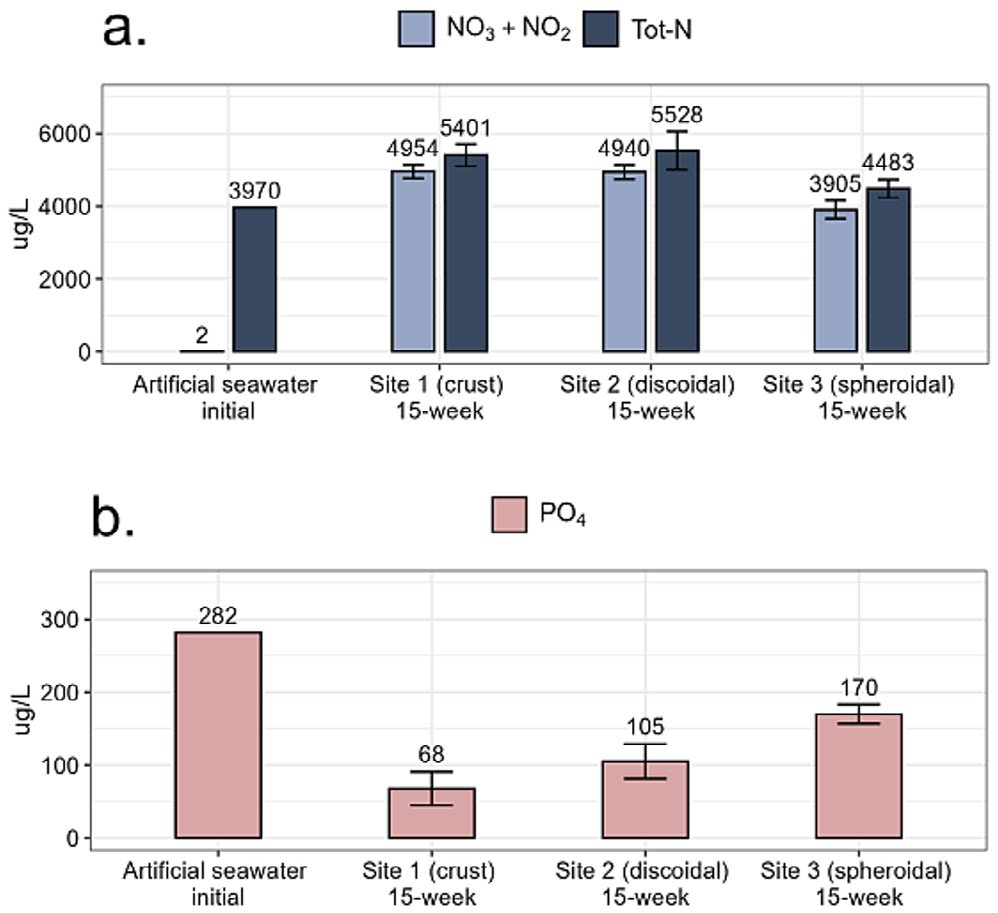
(a) Tot‐N, and NO_2_ + NO_3_, and (b) PO_4_^3−^, concentrations in artificial seawater and the mean concentrations in the 15‐week biotic triplicates (*n* = 3) from site 1 (crust), site 2 (discoidal), and site 3 (spheroidal). Error bars show the standard deviation in biotic triplicates. Data from the 0‐week microcosms or abiotic controls are not available.

The Tot‐N and NO_2_^−^ + NO_3_^−^ concentrations increased in the solution of biotic triplicates during 15 weeks of incubation, compared to the initial concentration in artificial seawater (Figure 8a and Table S12). The NO_2_^−^ + NO_3_^−^ increase was strong in all sites. In sites 1 (crust) and 2 (discoidal), Tot‐N and NO_2_^−^ + NO_3_^−^ increased the most, whereas in site 3 (spheroidal), the increase was smaller.

The PO_4_^3−^ concentration decreased in the incubation solution of biotic triplicates after 15 weeks, compared with the initial concentration in artificial seawater (Figure 8b and Table S12). In site 1, PO_4_^3−^concentration decreased the most, 4.1 times. In site 2, concentration decreased 2.7 times, and in site 3, it decreased 1.7 times, compared to the initial PO_4_^3‐^concentrations.

### 3.5 Intrinsic Microbial Community Composition on the Seabed and in Fe‐Mn Concretions From Microcosm Experiments

We received an average of 31 million read pairs (range: 6.4–49 million) from metagenomic sequencing and 52 million read pairs (range: 30–60 million) from metatranscriptomic sequencing, after sequence trimming, across fluffy, sediment, and Fe‐Mn concretion samples collected immediately after sampling (intrinsic) in 2022 and 2023. The mapping ratio of SSU rRNA read pairs was on average 0.05% in metagenomic samples and 37% in metatranscriptomic samples. Cells collected from the seawater by filtration had very low DNA and RNA concentrations, leading to failure in library preparation. Similarly, despite re‐extractions, sediment RNA concentrations were too low and failed in library preparation in the following samples: one replicate each from site 1 and site 3 in 2023, all replicates in site 2 in 2023, and all replicates in 2022 in all sites.

**FIGURE 9:**
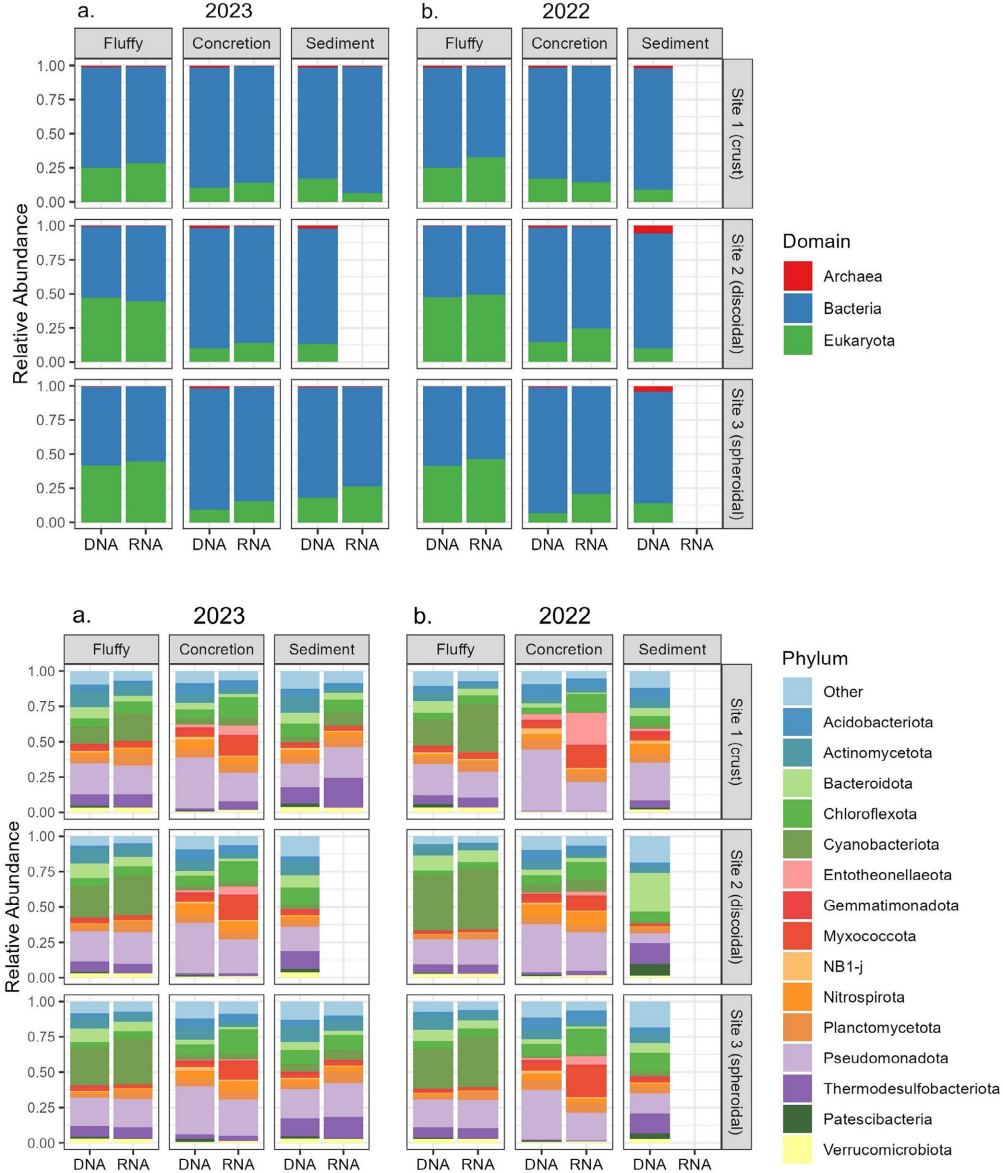
(upper panel) Relative abundances of archaea, bacteria, and eukaryota domains and (lower panel) the 15 most abundant archaeal and bacterial phyla across all intrinsic fluffy, Fe‐Mn concretion and sediment samples from each sampling site in year (a) 2023 and (b) 2022. SSU rRNA genes obtained from metagenomes are DNA and genes from metatranscriptomes are RNA. Biotic triplicates in RNA samples are presented as the mean relative abundance (*n* = 3, except for sediment samples in 2023, where *n* = 2, and Fe‐Mn concretion samples in 2022, where *n* = 1). Samples with missing data are presented as blank spaces. All other phyla not among the 15 most abundant are shown as the ‘Other’ group.

The SSU rRNA sequences obtained from metatranscriptomes (RNA, active microbial communities) and metagenomes (DNA, both active and dormant microbial communities) showed that bacteria were the most dominant (75%), followed by eukaryota (24%), and a small proportion of SSU reads belonged to the archaeal domain (1%) across fluffy, sediment, and intrinsic Fe‐Mn concretion samples in 2023 and 2022 (Figure 9; upper panel). Eukaryota were consistently more abundant in the fluffy samples compared to the sediments and concretions. The largest eukaryotic phyla were Amorphea and the SAR supergroups. At site 1 fluffy samples, approximately 25% of the SSU reads were eukaryotic, whereas at sites 2 and 3, nearly 50% were eukaryotic in both years. The remaining SSU reads were mainly assigned to bacteria, and fluffy samples had the least archaeal reads (0.6%). In Fe‐Mn concretions and sediments, eukaryal SSU reads were present at less than 25%. In both years, 85% of reads belonged to the bacterial domain across all Fe‐Mn concretion samples, and 83% in sediment samples. In concretions, archaeal SSU reads were detected around 1% of all samples. Archaea were most abundant in the sediment samples (2.3%), particularly in the 2022 samples.

The distribution of the 15 most abundant bacterial and archaeal phyla varied across sites in intrinsic fluffy, sediment, and Fe‐Mn concretion samples (Figure 9, lower panel). In fluffy samples across all sites, Cyanobacteriota was the most abundant phylum (28%), followed by Pseudomonadota (20%, previously Proteobacteria). Differences between the community data derived from DNA and RNA in fluffy samples were minor. Across all Fe‐Mn concretion samples, Pseudomonadota dominated both in DNA (36%) and RNA (23%) derived community. In DNA‐derived community, the next three largest phyla were Acidobacteriota (8%), Chloroflexota (6%), and Actinomycetota (6%). In RNA‐derived community, the next three largest phyla were Myxococcota (15%), Chloroflexota (15%), and Acidobacteriota (7%). In addition, site 1 concretions (crust) had a high abundance of Entotheonellaeota, particularly in RNA‐derived community in 2022, in which Entotheonellaeota formed 23% of relative abundance. The sediment samples showed most variation in phyla abundance and were predominantly dominated by Pseudomonadota (19%) and Thermodesulfobacteriota (13%). Additionally, in 2022, sediment from site 2 (discoidal) had a higher abundance of Bacteroidota (27%) than other sediment samples. Archaea were not among the 15 most abundant phyla. Communities obtained from DNA showed a higher proportion of members classified as ‘Other’ than those obtained from RNA. According to the Shannon diversity index (*H*′), the highest diversity was observed in sediment samples in 2023 (5.42 ± 0.55) and 2022 (5.59 ± 0.09), followed by Fe‐Mn concretions in 2023 (5.15 ± 0.18) and 2022 (4.94 ± 0.37). The lowest diversity was observed in fluffy samples in 2023 (4.67 ± 0.35) and 2022 (4.21 ± 0.32). ANOVA with Tukey HSD post hoc testing showed significantly higher Shannon diversity in concretion samples compared to fluffy samples (diff = 0.48, adj. *p* = 0.007), and sediment samples compared to fluffy samples (diff = 0.76, adj. *p* = < 0.001) in 2023. Sediment samples did not differ significantly from concretion samples. In 2022, Shannon diversity was significantly higher in concretion samples compared to fluffy samples (diff = 0.73, adj. *p* = 0.001), and significantly higher in sediment samples compared to both concretion samples (diff = 0.65, adj. *p* = 0.026) and to fluffy samples (diff = 1.37, adj. *p* = < 0.001). ANOVA assumptions of normality and homogeneity of variances were met (both *p* > 0.05). See Tables S13A and S13B for all Shannon diversity indexes, and Table S15A for ANOVA results.

**FIGURE 10:**
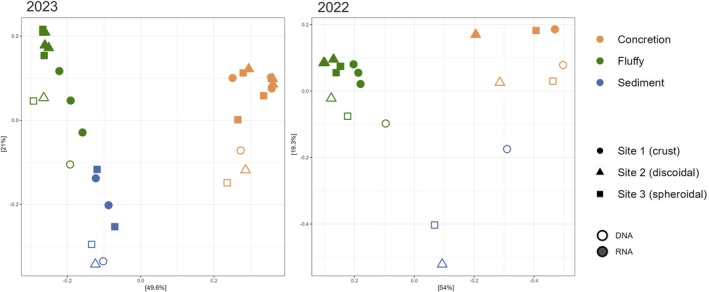
Principal Coordinate Analysis (PCoA) with Bray‐Curtis distance of microbial communities from intrinsic fluffy, Fe‐Mn concretion and sediment samples collected in year 2023 and 2022. DNA represents communities obtained from metagenomes, and RNA from metatranscriptomes. Community differences between each group (fluffy, sediment and Fe‐Mn concretion) were tested using PERMANOVA on Bray–Curtis distances. All pairwise comparisons among different groups were significant (adj. *p* ≤ 0.05). In 2023, sample sizes among each tested group were: Fluffy (*n* = 12), sediment (*n* = 7), and concretion (*n* = 12), and in 2022: fluffy (*n* = 12), sediment (*n* = 3), and concretion (*n* = 6). Raw sequence counts were normalised to relative abundances.

In 2023, the largest variation in the principal coordinate analysis (PCoA) ordination with Bray–Curtis distances was explained by 49.6% (PC1) and the second largest variation was explained by 21% (PC2) (Figure 10). In 2022, PC1 explained 54% and PC2 19.3% of the variation. In both years, samples clustered closely together based on their collected environment (fluffy, concretion, and sediment), while nucleic acid type (DNA/RNA) and sampling site had a minor influence on clustering. In 2023, fluffy samples from site 1 (crust) were distributed along a diagonal gradient from upper‐left to lower‐right, showing a slight clustering with sediment samples. In the year 2023, PERMANOVA indicated that community composition differed significantly between concretion and fluffy samples (*R* ^2^ = 0.66, adj. *p* = 0.001), concretion and sediment samples (*R* ^2^ = 0.54, adj. *p* = 0.001), and fluffy and sediment samples (*R* ^2^ = 0.46, adj. *p* = 0.001). The PERMANOVA assumption of homogeneity of multivariate dispersion was met (*p* > 0.05). In the year 2022, community composition differed significantly between concretion and fluffy samples (*R* ^2^ = 0.65, adj. *p* = 0.003), concretion and sediment samples (*R* ^2^ = 0.41, adj. *p* = 0.014), and fluffy and sediment samples (*R* ^2^ = 0.55, adj. *p* = 0.005). The PERMANOVA assumption of homogeneity of multivariate dispersion was not met (*p* < 0.05). See Table S16 for all *R* ^2^ and *p* values.

We received an average of 35 million read pairs (range: 8–111 million) from metagenomic sequencing and 53 million read pairs (range: 31–65 million) from metatranscriptomic sequencing, after sequence trimming, across all Fe‐Mn concretion samples collected from incubation experiments in 2023 and 2022. Mapping ratio of SSU rRNA read pairs was on average 0.04% for metagenome samples and 37% for metatranscriptome samples. One 0‐week replicate from the biotic triplicates at site 3 in 2022 failed library preparation. We received total RNA concentrations from abiotic controls in year 2022 from site 1, 40 ng/uL (0‐week), 10 ng/uL (12‐week), and site 2, 15 ng/uL (0‐week), and from year 2023 experiment from site 1, 10 ng/uL (0‐week), and site 2, 10 ng/uL (0‐week) and 7 ng/uL (15‐week).

**FIGURE 11:**
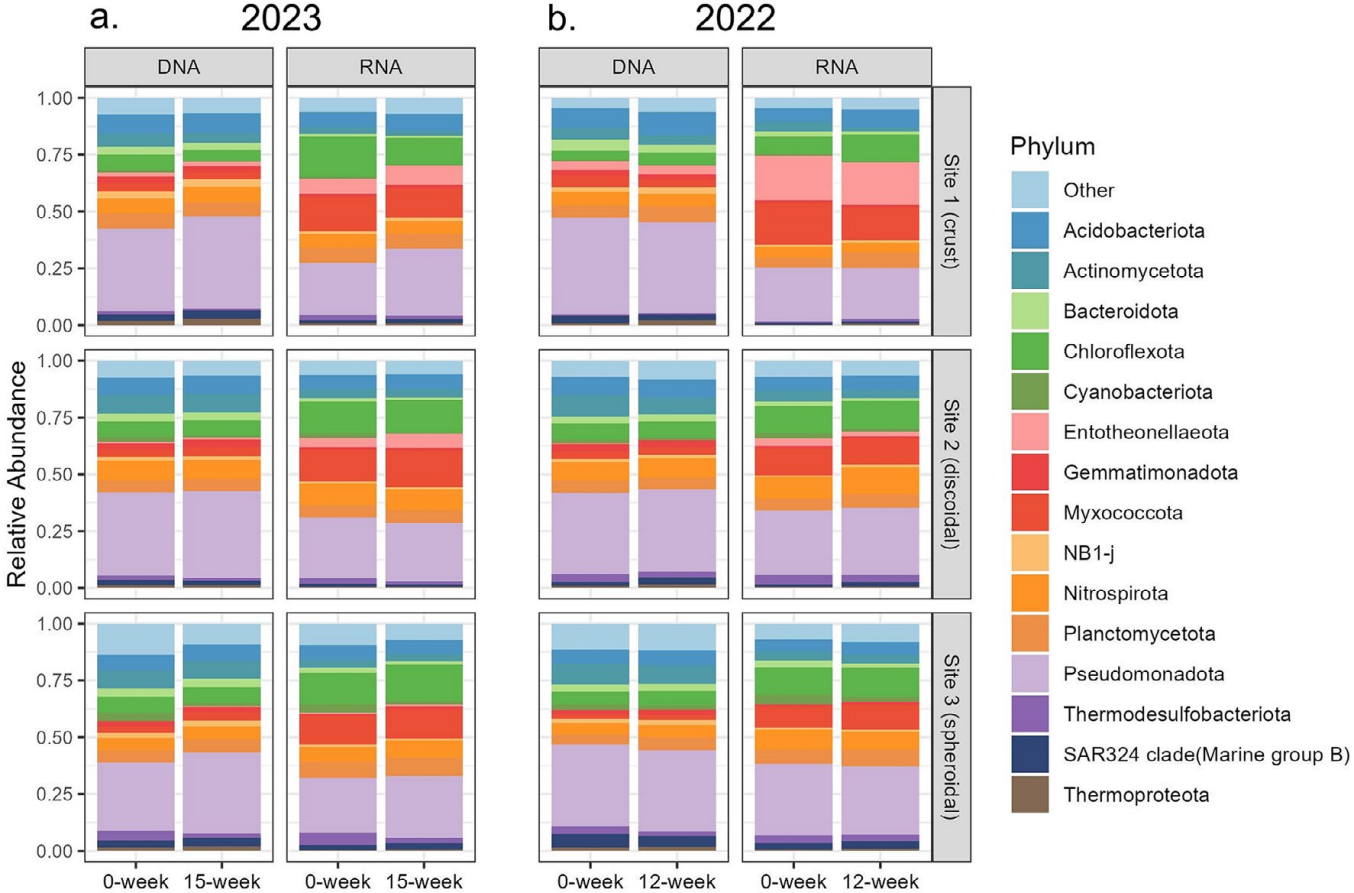
Relative abundances of the 15 most abundant bacterial and archaeal phyla across all Fe‐Mn concretion samples collected from biotic triplicates of microcosm experiments in year (a) 2023 and (b) 2022. DNA represents SSU rRNA genes obtained from metagenomes and RNA from metatranscriptomes. Biotic triplicates are presented as the mean relative abundance from DNA and RNA (*n* = 3, except for the 2022 site 3 0‐week RNA‐derived samples, where *n* = 2). All other phyla that did not belong to the 15 most abundant are shown as the ‘Other’ group.

When comparing SSU rRNA sequences obtained from metatranscriptomes (RNA) with sequences obtained from metagenomes (DNA), the 15 most abundant bacteria and archaea across Fe‐Mn concretions collected from microcosm incubation experiments had very similar profiles in all sites in both 2023 and 2022 (Figure 11). Across all concretion samples, Pseudomonadota was the most abundant bacterial phylum (32%). Its abundance was higher in DNA‐derived communities than in RNA‐derived communities. Other large groups were Chloroflexota (10%), Myxococcota (8%), Nitrospirota (7%), Acidobacteriota (7%), and Planctomycetota (6%). Myxococcota, Chloroflexota, and Nitrospirota had higher relative abundances in RNA‐derived communities compared to DNA‐derived communities. Phylum Entotheonellaeota was abundant in site 1 samples (crust), both years, but particularly in 2022. Unlike in intrinsic Fe‐Mn concretions (Figure 9, lower panel), SAR324 clade and Thermoproteota (archaea) were among the 15 most abundant phyla in concretions collected from incubation experiments. The occurrence of the archaeal phyla was low, and the phylum Thermoproteota was present in low abundances (1%) across all samples. The following three largest archaeal phyla were Asgardarchaeota, Nanoarchaeota, and Halobacteriota. They were detected at low abundance (< 1%). A community composition obtained from DNA showed a slightly higher proportion of the community classified as ‘Other’ than in RNA samples.

**FIGURE 12:**
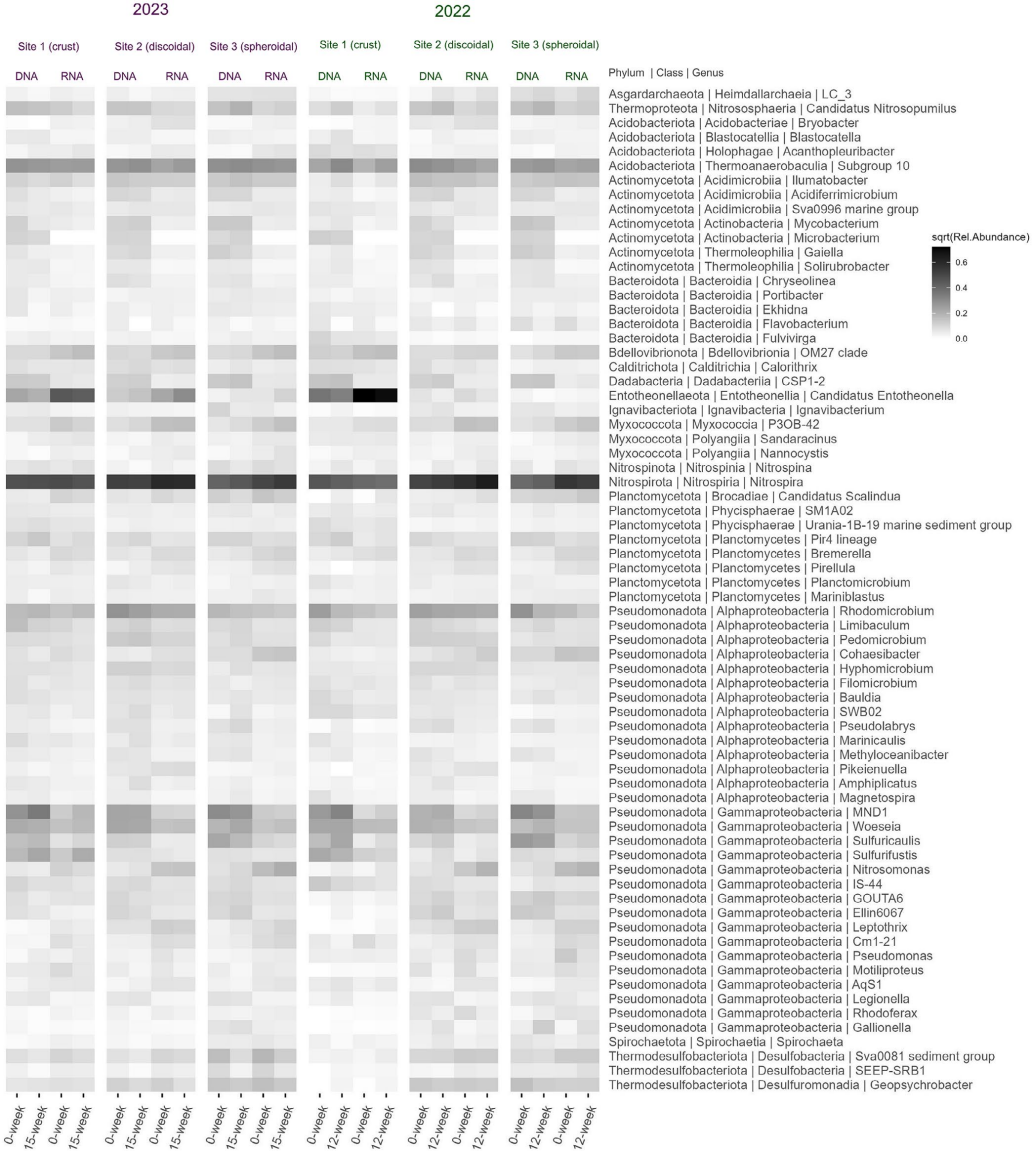
Relative abundances of the 70 most abundant bacterial and archaeal genera across all Fe‐Mn concretion samples collected from biotic triplicates from incubation experiments in 2023 and 2022. The heatmap is arranged alphabetically by domain, phylum, and class. Two archaeal genera are at the top. The most abundant genus within each class is positioned at the top of each class. DNA represents SSU rRNA genes obtained from metagenomes and RNA from metatranscriptomes. Biotic triplicates are presented as the mean abundance from DNA and RNA (*n* = 3, except for the 2022 site 3 0‐week RNA‐derived samples, where *n* = 2). Abundances are presented as the square root of relative abundances.

From the 70 most abundant archaeal and bacterial genera in Fe‐Mn concretions from incubation experiments (Figure 12), the 10 most abundant across all samples were *Nitrospira* (phylum: Nitrospirota, with relative abundances ranging from 15% to 41% of total community), *Subgroup 10* (Acidobacteriota, 3.7%–9.4%), *Candidatus Entotheonella* (Entotheonellaeota, 0.03%–52%), *MND1* (Pseudomonadota, 0.8%–12.5%), *Rhodomicrobium* (1.6%–8.1%, Pseudomonadota), *Woeseia* (1.9%–5.3%, Pseudomonadota), *Sulfuricaulis* (0.2%–7.0%, Pseudomonadota), *Candidatus Nitrosopumilus* (0.3%–3.9%, Thermoproteota, Archaea), *Ilumatobacter* (0.1%–2.8%, Actinomycetota), and *OM27 clade* (0.5%–2.9%, Bdellovibrionota). Classes Alpha‐ and Gammaproteobacteria (Pseudomonadota) formed the largest part of the community. Major differences between the 0‐week and respective 12‐ or 15‐week community abundance were not detected. *Candidatus Entotheonella* (Entotheonellaeota) was abundant mainly in site 1 (crust) samples in both years, and it also showed higher counts in RNA‐derived than in DNA‐derived communities. Furthermore, in site 1 RNA‐derived samples, particularly in year 2022, many genera in Class Gammaproteobacteria (from GOUTA6 to Gallionella), genus Spirochaeta (Spirochaetota) and genera Sva0081 sediment group, SEEP‐SRB1, and Geopsychrobacter (Thermodesulfobacteriota) received lower abundance compared to the respective site 2 (discoidal) and 3 (spheroidal) RNA‐derived samples. The DNA‐derived community received generally higher abundances across all samples and genera. According to the Shannon diversity index (*H*′), the highest diversity was observed in site 3 (spheroidal) concretions in 2023 (5.35 ± 0.13) and 2022 (5.36 ± 0.11), followed by site 2 (discoidal) concretions in 2023 (5.15 ± 0.13) and 2022 (5.27 ± 0.08). The lowest diversity was observed in site 1 (crust) concretions in 2023 (5.06 ± 0.17) and 2022 (4.62 ± 0.31). ANOVA with Tukey HSD post hoc testing showed significantly higher Shannon diversity in spheroidal morphotype compared to crusts (diff = 0.29, adj. *p* = < 0.001) and discoidal in 2023 (diff = 0.20, adj. *p* = 0.006). No significant differences were found between discoidal and crust morphotypes. ANOVA assumptions of normality and homogeneity of variances were met (both *p* > 0.05). In 2022 samples, Welch's ANOVA followed by Games‐Howell post hoc test showed significantly higher Shannon diversity in spheroidal morphotype compared to crusts (diff = 0.74, adj. *p* = < 0.001), and discoidal compared to crusts (diff = 0.65, adj. *p* = < 0.001). No significant differences were found between spheroidal and discoidal morphotypes. See Table S14 for all Shannon diversity indexes, and Table S15B for ANOVA with post hoc results.

**FIGURE 13:**
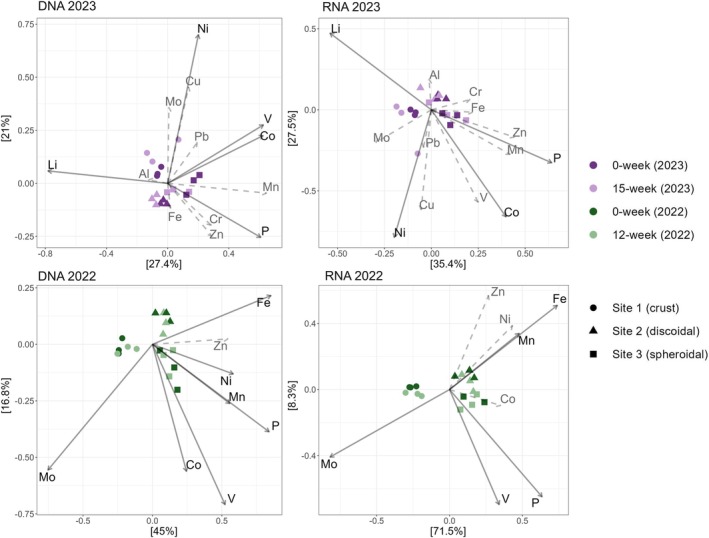
Principal coordinate analysis (PCoA) with Bray‐Curtis distance of archaeal and bacterial communities from Fe‐Mn concretions collected from incubation experiments in 2023 and 2022. DNA represents communities obtained from metagenomes, and RNA from metatranscriptomes. Community differences between each site/morphotype (crust, discoidal, spheroidal) were tested using PERMANOVA on Bray–Curtis distances. All pairwise comparisons among different sites were significant (adj. *p* ≤ 0.05). Sample sizes among each tested site were *n* = 6, except for the 2022 site 3 RNA‐derived samples, where *n* = 5. Arrow directions indicate the gradient of each element concentration in ordination space, while arrow lengths reflect the strength of correlation with microbial community composition. Solid grey arrows show significant correlations (adj. *p* ≤ 0.05), and dashed grey arrows indicate non‐significant to illustrate potential associations with community composition. Raw sequence counts were normalised to relative abundances. Element concentrations were first normalised to measured values of artificial seawater (Table S2) and then log‐transformed.

In DNA‐derived communities in 2023, the largest variation in bacterial and archaeal community composition was explained by 27.4% (PC1), and the second largest variation by 21% (PC2) in the Principal Coordinate Analysis (PCoA) ordination with Bray‐Curtis distances (Figure 13). In RNA‐derived communities, PC1 explained 35.4% and PC2 27.5% of the variation. In both DNA and RNA‐derived communities, samples clustered together based on their morphotype (crust, discoidal, and spheroidal), rather than time of collection (0‐week or 12/15‐week). PERMANOVA on Bray–Curtis dissimilarities showed that all three sites (morphotypes) differed significantly from each other and that morphotype explained 30%–40% of the variation in community structure. In DNA‐derived communities, community composition differed significantly between crust and discoidal (*R* ^2^ = 0.31, adj. *p* = 0.008), crust and spheroidal (*R* ^2^ = 0.29, adj. *p* = 0.003), and discoidal and spheroidal morphotypes (*R* ^2^ = 0.31, adj. *p* = 0.003). In RNA‐derived communities, community composition differed significantly between crust and discoidal (*R* ^2^ = 0.41, adj. *p* = 0.004), crust and spheroidal (*R* ^2^ = 0.37, adj. *p* = 0.004), and discoidal and spheroidal morphotypes (*R* ^2^ = 0.36, adj. *p* = 0.004). The PERMANOVA assumption of homogeneity of multivariate dispersion was met (*p* > 0.05).

In DNA‐derived communities in 2023, the majority of elements (Fe, Cr, Zn, P, Mn Co and V) oriented toward site 3 (spheroidal), and Cu, Ni, Mo, Al and Li oriented toward site 1 (crust). Pb was positioned between clusters and did not show a clear association with any samples. Statistical testing showed that P (*R* ^2^ = 0.44, adj. *p* = 0.05) Co (*R* ^2^ = 0.44, adj. *p* = 0.05), V (*R* ^2^ = 0.47, adj. *p* = 0.03), Ni (*R* ^2^ = 0.53, adj. *p* = 0.03), and Li (*R* ^2^ = 0.62, adj. *p* = 0.03) correlated significantly with the ordination. RNA‐derived communities showed a similar pattern, in which most elements (Cr, Fe, Zn, P, Mn Co and V) oriented toward site 3 (spheroidal), Al toward site 2 (discoidal), and Mo, Pb, Ni and Cu toward site 1 (crust). Li did not show a clear association with any samples. The Co (*R* ^2^ = 0.59, adj. *p* = 0.02), P (*R* ^2^ = 0.52, adj. *p* = 0.04), Li (*R* ^2^ = 0.51, adj. *p* = 0.03), and Ni (*R* ^2^ = 0.66, adj. *p* = 0.02) correlated significantly with the ordination. The elements that were not statistically significant (adj. *p* ≤ 0.05) illustrate their strength and the potential associations with community composition. See Table S17 for all *R* ^2^ and *p* values.

In DNA‐derived communities in 2022, PC1 explained 45% of the variation in bacterial and archaeal community composition, and PC2 explained 16.8%. In RNA‐derived communities, PC1 explained 71.5% and PC2 explained 8.3% of the variation. In both DNA‐ and RNA‐derived communities, samples clustered together based on their morphotype (crust, discoidal, and spheroidal), rather than time of collection. PERMANOVA on Bray–Curtis dissimilarities showed significant differences among all sites (morphotypes), with the strongest separation in crust vs. discoidal and crust vs. spheroidal morphotypes. In DNA‐derived communities, community composition differed significantly between crust and discoidal (*R* ^2^ = 0.56, adj. *p* = 0.006), crust and spheroidal (*R* ^2^ = 0.54, adj. *p* = 0.005), and discoidal and spheroidal morphotypes (*R* ^2^ = 0.30, adj. *p* = 0.005). In RNA‐derived communities, community composition differed significantly between crust and discoidal (*R* ^2^ = 0.75, adj. *p* = 0.005), crust and spheroidal (*R* ^2^ = 0.76, adj. *p* = 0.005), and discoidal and spheroidal morphotypes (*R* ^2^ = 0.28, adj. *p* = 0.005). The PERMANOVA assumption of homogeneity of multivariate dispersion was met (*p* > 0.05).

In DNA‐derived communities in 2022, most elements (Zn, Ni, Mn, P, V, and Co) oriented toward site 3 (spheroidal), Fe toward site 2 (discoidal), and Mo toward site 1 (crust). Statistical testing showed that P (*R* ^2^ = 0.86, adj. *p* = 0.002), Fe (*R* ^2^ = 0.78, adj. *p* = 0.002), V (*R* ^2^ = 0.78, adj. *p* = 0.002), Mn (*R* ^2^ = 0.38, adj. *p* = 0.04) Co (*R* ^2^ = 0.38, adj. *p* = 0.03), Ni (*R* ^2^ = 0.36, adj. *p* = 0.05), and Mo (*R* ^2^ = 0.88, adj. *p* = 0.002) correlated significantly with the ordination. In RNA‐derived communities, V, P, and Co oriented toward site 3 (spheroidal) and Mn, Fe, Ni, and Zn toward site 2 (discoidal), whereas Mo showed a trend toward site 1 (crust). The P (*R* ^2^ = 0.82, adj. *p* = 0.002), Fe (*R* ^2^ = 0.81, adj. *p* = 0.002), V (*R* ^2^ = 0.60, adj. *p* = 0.002), Mo (*R* ^2^ = 0.84, adj. *p* = 0.002), and Mn (*R* ^2^ = 0.34, adj. *p* = 0.05) correlated significantly with the ordination. The elements that were not statistically significant (adj. *p* ≤ 0.05) illustrate their strength and the potential associations with community composition. See Table S17 for all *R* ^2^ and *p* values.

## 4 Discussion

The formation of Fe‐Mn concretions is a microbially facilitated biogeochemical process where Fe and Mn from seawater and sediment porewater precipitate to form (hydr)oxides on the seafloor (e.g., Winterhalter 2004). The formation process requires predominantly oxygenated conditions and no or low sedimentation rates (e.g., Winterhalter 2004; Zhamoida et al. 1996). At all three study sites, bottom water was well‐oxygenated (> 6 mL/L) at the time of sampling (Figure 3). The concretions occurred as a ca. 2 cm thick layer on the seafloor, covering exposed old fine‐grained sediments from the previous lacustrine phase of the Baltic Sea Basin, which have lower organic matter contents and are thus more favourable for the Fe‐Mn precipitation than the reducing organic‐rich mud that is being deposited in the Baltic Sea today (Virtasalo et al. 2007, 2014). At all three sampling sites, the conditions were therefore favourable for the formation of ferromanganese concretions.

The microbial community composition of intrinsic Fe‐Mn concretions differed significantly from that in the surface organic‐rich ‘fluffy’ mud layer and surface sediment (Figures 9 and 10), indicating that Baltic Sea concretions are a highly selective environment for microbes, consistent with observations from deep‐sea polymetallic nodules (Blöthe et al. 2015; Tully and Heidelberg 2013; Molari et al. 2020). However, because all RNA sediment samples from 2022 did not yield usable data, interannual comparisons were not possible, and the reduced sample size limited statistical power and the robustness of these observations.

The community composition in intrinsic Fe‐Mn concretions and in those collected from incubation experiments was similar, with bacteria comprising most of the community and archaea present at low abundances (Figures 9 and 11). Phylum Pseudomonadota dominated the communities, followed by Chloroflexota, Acidobacteriota, Myxococcota (previously Deltaproteobacteria), and Nitrospirota, resembling previously described compositions for Fe–Mn concretions in the Gulf of Finland (Yli‐Hemminki et al. 2014; Reunamo et al. 2017). While Pseudomonadota dominated in total sequence numbers, their abundance was distributed across multiple genera, having a more diverse species community composition in Fe‐Mn concretions (Figure 12). Furthermore, a smaller proportion of Pseudomonadota within the total community was active (as indicated by RNA), suggesting that only a subset of Pseudomonadota actively participates in metabolic processes. A small proportion (3%–4%) was assigned to ‘Other’, representing communities with rare or low abundance. Although this rare biosphere is present at low abundances, it is increasingly recognised that these taxa can influence ecological roles and possess metabolic potential to support overall ecosystem functioning and stability (Zhang et al. 2021; Jousset et al. 2017), and the same may apply to communities in Fe‐Mn concretions.

### 4.1 Crust, Discoidal and Spheroidal Fe‐Mn Concretion Morphotypes Host Different Microbial Communities and Metal Accumulation Patterns

We did not observe major differences between the 0‐week and the respective 12‐ or 15‐week active microbial community compositions (Figures 11 and 12), showing that incubation did not strongly select for different taxa and that Fe‐Mn concretions have relatively stable communities. Instead, community composition was significantly associated with Fe‐Mn concretion morphology (Figure 13). In the experiments with both discoidal and spheroidal Fe‐Mn concretion morphotypes, dissolved metal concentrations in the surrounding artificial seawater tended to be lower in biotic triplicates than in the respective abiotic controls (Figure 5), indicating that metals in artificial seawater were precipitated more efficiently as a new Fe‐Mn concretion material when microbes were present in these two morphotypes. Metals could have been incorporated into Fe‐Mn concretions or precipitated onto bottle walls, or both. Microbes enhanced metal accumulation most in experiment with spheroidal morphotype, in which metals (Mn, Ni, V, Mo, Al, Cr and Pb) were removed from surrounding solution (Figure 5) and many metals (Mn, Fe, Al Co, Ni, V, Zn, Mo, Cr, Cu, and Pb) were precipitated more efficiently on the bottle walls in presence of microbes (Figure 6). In the discoidal morphotype metal precipitation on the bottle walls was not enhanced by microbes. Similar results were reported in our previous study, done in 2022 (Majamäki et al. 2025), which also showed that microbial activity enhanced metal accumulation most substantially in discoidal and spheroidal morphotypes. Several metals, including Fe, Mn, V Co, Zn, and Cr oriented toward the spheroidal and discoidal morphotypes (Figure 13), suggesting that microbial communities in these concretion types are associated with elevated concentrations of these metals and that community composition varies along metal gradients. A similar pattern was observed in the 2022 experiment (Figure 13). Together with results obtained in this study and from previous experiment (Majamäki et al. 2025), we demonstrate elevated metal accumulation by microbes in discoidal and spheroidal morphotypes. It is important to note though that only a single abiotic control and three biotic treatments were used per morphotype, which limits statistical strength of biotic‐abiotic comparisons. Furthermore, NaN_3_ that was used in abiotic controls to inhibit microbial activity (Cabrol et al. 2017; Sujith et al. 2017) can alter the chemical composition and redox state of different compounds, producing unwanted by‐products (Hendrix et al. 2019). Although these unwanted reactions were mainly observed in longer incubation experiments (years), short‐term (minutes) effects may also occur, influencing here the reduction of Fe^3+^ and formation of N_2_O (Figure 4). Therefore, while NaN_3_ is effective for inhibiting microbial activity, any potential effects of NaN_3_ should be acknowledged when interpreting the results.

Synchrotron XRF and XAS microanalyses document that discoidal and spheroidal morphotypes in the Baltic Sea contain alternating Mn‐(hydr)oxide‐rich and Fe‐(hydr)oxide‐rich layers, whereas crust concretions are predominantly composed of Fe‐(hydr)oxides (Wasiljeff, Yu, et al. 2025). Mn‐rich layers exhibit columnar and branched dendritic growth structures, interpreted to result from microbially induced precipitation. Local environmental factors, such as hydrodynamics and sediment depositional conditions on the seafloor, influence the Fe‐Mn concretion morphotype and their characteristic geochemical compositions (Winterhalter 2004; Zhamoida et al. 1996; Wasiljeff, Yu, et al. 2025). In addition, morphology and composition may be influenced by intermittent burial of the Fe‐Mn concretions by the organic‐rich ‘fluffy’ mud layer (Wasiljeff, Yu, et al. 2025; Shulga et al. 2022). The fluffy mud layer transports organic matter, Fe, and Mn, and detrital minerals (Hlawatsch, Neumann, et al. 2002; Wasiljeff, Yu, et al. 2025) and may promote transient anoxia, under which Mn phases preferentially dissolve, enhancing the incorporation of detrital minerals into Fe‐(hydr)oxides and favouring the formation of Fe‐rich layers (Wasiljeff, Yu, et al. 2025; Shulga et al. 2022).

We hypothesise that these environmental conditions control Fe‐Mn concretion morphology and geochemical composition, and these factors together influence microbial community composition. Distinct morphotypes likely provide different mineralogical substrates and redox conditions for microbes. Consequently, differences in microbial community composition we observed in our data may reflect direct responses to concretion morphology, mineralogy and geochemistry, the environmental conditions that give rise to these characteristics, or interactions between all these factors. Furthermore, microbial communities may in turn regulate element cycling and concretion growth by oxidising and reducing metals (e.g., Yli‐Hemminki et al. 2014; Sujith et al. 2017), likely shaping mineralogical and elemental composition of Fe‐Mn concretions and potentially contribute to morphotype‐specific metal accumulation patterns (Figure 5; Majamäki et al. 2025). However, our results do not allow us to determine conclusively whether microbial communities respond to Fe‐Mn concretion morphology and geochemistry or actively contribute to shaping them.

Class Gammaproteobacteria (Pseudomonadota) received higher relative abundance in the discoidal and spheroidal morphotypes than in the crust morphotype, particularly in 2022, based on RNA‐derived active microbial communities (Figure 12). Within this class, we observed several genera capable of oxidising Fe and Mn, such as *Gallionella*, *Leptothrix*, and *Pseudomonas*, highlighting their potential contribution to the enrichment of Fe and Mn in concretions. Higher abundance of Gammaproteobacteria in discoidal and spheroidal morphotypes may have therefore facilitated elevated metal accumulation in these two morphotypes. For example, *Gallionella* sp. and *Leptothrix* sp. include well‐known Fe‐oxidising bacteria, commonly found in environments rich in Fe (Hallbeck and Pedersen 2015; Tothero et al. 2024). In addition, some *Leptothrix* sp. and *Pseudomonas* sp. have the potential to oxidise Mn (Adams and Ghiorse 1986; Nelson et al. 1999; Wright et al. 2018; Geszvain et al. 2013; Ghiorse 1984) with the latter previously identified in both Mn‐oxidising and Fe‐oxidising experiments conducted on Fe‐Mn concretions of the Gulf of Finland (Yli‐Hemminki et al. 2014), showing their consistent presence in Fe‐Mn concretions and potential contribution to metal cycling. Oxidation of Fe or Mn may also indirectly affect other metal cycling. For example, Mn oxidisers can indirectly promote greater Cu accumulation since Cu binds strongly to Mn (hydr)oxides by occupying vacancy sites in birnessite (δ‐MnO2) (Sherman and Peacock 2010). The lower abundance of potential Mn oxidisers within class Gammaproteobacteria in the crust morphotype may therefore explain the highest Cu dissolution.

In addition to Gammaproteobacteria, sulfate‐reducing bacteria (SRB), such as Sva0081 sediment group and SEEP‐SRB1 (Desulfobacteria), and Fe‐reducer *Geopsychrobacter (*Desulfuromonadia) showed higher relative abundances in discoidal and spheroidal morphotypes, compared to crusts among active microbial communities (Figure 12). Because concretions are highly porous (Wasiljeff et al. 2024; Wasiljeff, Yu, et al. 2025), local anoxic conditions can develop within their pores and on their surfaces, where facultative anaerobes or anaerobes, such as SRB, can thrive (Muyzer and Stams 2008). In contrast, we also identified sulfur‐oxidising bacteria (SOB), such as *Sulfuricaulis* and *Sulfurifustis* (Gammaproteobacteria), in all morphotypes. The presence of both anaerobic and aerobic bacteria demonstrates the occurrence of microscale oxic‐suboxic‐anoxic zonations within concretions and that sulfur is continuously reduced and re‐oxidised. Higher abundance of SRB in discoidal and spheroidal concretions could be explained by their more porous structure, arising from columnar and branched dendritic growth texture in Mn‐rich phases (Wasiljeff et al. 2024; Wasiljeff, Yu, et al. 2025), likely to provide greater microhabitat diversity for microbes and further enhance formation of anoxic microniches. Given that, active SRB abundances decreased slightly after incubation, while SOB abundance increased, suggesting SOB dominance in discoidal and spheroidal morphotypes. This may indirectly affect Fe concentrations in solution, since SOB oxidises sulfides such as HS^−^ and H_2_S, which can limit Fe(II) precipitation as FeS. This could maintain higher dissolved Fe concentrations in the surrounding artificial seawater, which could partly explain increased Fe concentrations in both morphotypes (Figure 5). However, although SOB clearly dominated in the crust morphotype, it was not attributed to an exceptionally high increase of Fe in solution.

Only the crust morphotype was associated with a high relative abundance of the active Entotheonellaeota in both intrinsic concretions and those collected from incubation experiments (Figures 9, 10 and 12). *Candidatus Entotheonella* is a filamentous bacterium that is best known for its symbiotic association with marine sponges, possessing a unique set of biosynthetic genes and the potential to produce bioactive natural products (Kim et al. 2025; Lackner et al. 2017; Dell et al. 2026; Yamabe et al. 2024), where it acts as a detoxifying organ for the sponge, protecting it by sequestering arsenic and barium (Keren et al. 2017). Previously, Entotheonellaeota has been characterised from shallow groundwater wells in Finland (Lyons et al. 2021) and from deep‐sea polymetallic nodules and sediments (Molari et al. 2020). It is suggested that *Entotheonella* sp. has intracellular spheres rich in Mo, indicating that the bacteria actively sequester and accumulate the element (Shoham et al. 2024). Although we did not observe an exceptionally high accumulation of Mo in the crust morphotype in this study (Figures 5 and 6) or in the previous study (Majamäki et al. 2025), Mo oriented toward crust samples and correlated strongly with community composition in 2022, in which the highest abundance of active *Candidatus Entotheonella* was detected. This suggests that Mo is an important factor associated with communities in the crusts, and the potential role of *Entotheonella* sp. in the accumulation of Mo in crust Fe‐Mn concretions.

### 4.2 Several Fe‐ and Mn‐Oxidising and Reducing Bacteria Potentially Contributing to Metal and REE Cycling in Fe‐Mn Concretions

In addition to several Fe and Mn oxidisers observed in class Gammaproteobacteria, other potential players in the Fe or Mn oxidation were observed among Alphaproteobacteria, such as *Hyphomicrobium*, *Pedomicrobium*, and *Filomicrobium* (Figure 12). They share similar morphology and ecological niches by reproducing by budding and forming hyphae (Hirsch 2015). In particular, *Pedomicrobium* sp. can oxidise Fe(II) or Mn(II), converting them into (hydr)oxides that precipitate and accumulate extracellularly around the cells. In contrast to these metal oxidisers, we identified genera capable of reducing Fe and Mn, such as *Rhodoferax* and *Acidiferrimicrobium* (Figure 12) (Finneran et al. 2003; González et al. 2020). Together, these taxa indicate potential for both Fe and Mn oxidation and reduction, suggesting their role in metal cycling in Fe‐Mn concretions and further confirming redox heterogeneity in concretions. Furthermore, *Magnetospira* (Alphaproteobacteria) in our samples (Figure 12) belongs to a unique group of magnetotactic bacteria (MTB) that synthesise magnetite (Fe_3_O_4_) crystals intracellularly and use them to navigate magnetic fields (Bazylinski and Frankel 2004; Williams et al. 2012). As a result, they accumulate Fe from the surrounding environment (Amor et al. 2020) and may thus indirectly influence the Fe cycle within concretions. Previously, spheroidal concretions from the Baltic Sea have been associated with enhanced microbial biomineralisation rates and magnetofossil content, produced by MTB (Wasiljeff et al. 2024).

In the beginning of the experiment (0‐week), REY patterns in all incubations showed a subtle but distinct MREE bulge, suggesting the REY originates from reductive dissolution of Fe‐Mn (oxyhydr)oxide solid phases (Figure 7) (Haley et al. 2004; Abbott et al. 2015). Since REY were not introduced to the artificial seawater incubation solutions at the initial stage of the experiment, they must originate from the Fe‐Mn concretions. It is likely the REY and the observed MREE enrichment pattern originated from porewaters of the concretion internal pore networks, or they were partially desorbed from surface‐adsorbed Fe and Mn complexes due to interfacial redox reactions and minor changes in pH shortly after the concretion material had been introduced into the microcosms (Majamäki et al. 2025). The REY patterns remained mostly similar in solution of both the biotic triplicates and abiotic controls while exhibiting differences among the distribution of REYs during the experiment. In general, the LREE are more particle reactive, having shorter residence time in solution than HREE, which could explain the preferential removal of LREE over HREE from the solution during the experiment. Material that precipitated on the bottle walls during incubation showed consistently higher REE concentrations in the biotic triplicates, suggesting that REE precipitation was enhanced in the presence of microbes. As discussed above, REEs were likely released from the Fe‐Mn concretions and subsequently incorporated into newly formed Fe‐Mn (hydr)oxides on the bottle walls or were scavenged back into the concretions. The bottle walls likely provided well‐oxygenated surfaces that favoured microbial oxidative metabolism and the formation of fresh Fe and Mn (hydr)oxides produced either abiotically or microbially through Fe and Mn oxidation, which could capture REEs efficiently (Bau and Koschinsky 2009; Liu et al. 2018; Sjöberg et al. 2017, 2018).

In natural environments, Fe and Mn (oxyhydr)oxides preferentially scavenge MREE from the surrounding water column that is then mobilised in early diagenesis (Surya Prakash et al. 2012; Deng et al. 2022). After the 15‐week incubations, approximately linear REY patterns across all samples, which showed nearly constant increase from LREE to HREE, resemble the patterns commonly obtained in oxic brackish water and seawater (e.g., Sholkovitz et al. 1992; Molina‐Kescher et al. 2014). Our data further showed a positive correlation between Mn (Fe) and LREE (HREE) and a negative correlation with Mn (Fe) and HREE (LREE), which aligns with previous research on marine Fe‐Mn deposits indicating preferential sorption of HREE to Fe phases (Jiang et al. 2011; Szamałek et al. 2018). The dissolution of Fe during the experiment and precipitation of Mn reflect these intrinsic complexations. The decrease in Ce concentration in the solution during the 15‐week experiment and the subtle negative Ce anomalies observed in the REY patterns could reflect the oxidative scavenging of Ce to the precipitates, resulting in a negative anomaly in the solution (Piper 1974; Ingri and Pontér 1986; Dubinin 2004; Bau and Koschinsky 2009). This is not evident, however, since there is no observable Ce anomaly in the newly formed precipitates after the 15‐week experiment. In the presence of microbes and/or organic compounds, an absence of solid phase Ce anomaly has been previously reported (Kraemer et al. 2017). The binding of REE with humate ligands effectively inhibits the formation of Ce anomalies during the partitioning of REE between the surrounding solution and the precipitates (Kraemer et al. 2017). The positive Eu anomaly in solution and negative Eu anomaly in the precipitate REE patterns likely imply the strong preferential complexation with carbonate ions in the artificial seawater (Censi et al. 2015).

### 4.3 Fe‐Mn Concretions Hosted Archaea and Bacteria, Contributing to the Nitrogen and Methane Cycles

Headspace CO_2_ increased strongly in all biotic triplicates, showing microbial respiration and activity in all three Fe‐Mn concretion morphotypes (Figure 4). The slight increase in CO_2_ concentrations observed in abiotic controls may result from the low concentration of added NaN_3_ (50 mM) and the extended incubation period, which may have allowed minimal microbial activity (Cabrol et al. 2017). Alternatively, this small increase may have resulted from the addition of NaN_3_ which can cause abiotic reactions such as the reduction of functional groups in dissolved organic matter (Hendrix et al. 2019). The decrease in methane across all biotic triplicates indicated potential microbial consumption after 15 weeks of incubation, most likely driven by aerobic methanotrophy, as also suggested in the 2022 experiment (Majamäki et al. 2025). Furthermore, the greatest decrease in methane was observed with the crust concretion morphotype, as in 2022. The genus *Methyloceanibacter*, observed in all our samples (Figure 12), includes species that can utilise one‐carbon compounds other than methane, although the *Methyloceanibacter* strain was also found to directly oxidise methane using a soluble methane monooxygenase (Vekeman et al. 2016). Microcosms containing sulfate, Fe, Mn, nitrate and nitrite can also support anaerobic methane oxidation (AOM), which typically occurs by consortia with methane‐oxidising ANME archaea and bacteria that reduce the respective electron acceptors (Orphan et al. 2001; Leu et al. 2020). However, the abundance of ANME archaea (belonging to the phylum Halobacteriota) was very low in our samples (< 1%), implying that AOM is unlikely to contribute substantially to methane oxidation in microcosms, suggesting that the methane decrease is primarily the result of aerobic methane oxidation.

Nitrous oxide was produced excessively in crust‐shaped concretions during incubation and also in small amounts in discoidal and spheroidal morphotypes in biotic triplicates, indicating the presence of denitrifies (Figure 4). *Pseudomonas* (Gammaproteobacteria), in our samples (Figure 12), include well‐known denitrifies (Zumft 1997), such as *P. aeruginosa* , which can perform a complete denitrification cycle (Carlson and Ingraham 1983). Furthermore, *Woeseia* (Gammaproteobacteria) has species with the potential for partial denitrification pathways (Mußmann et al. 2017) and were previously identified from oceanic nodules (Shulga et al. 2022; Molari et al. 2020; He et al. 2025; Tominaga et al. 2023). Concentrations of nitrate and nitrite and total nitrogen increased in the solution of biotic triplicates (Figure 8) after 15 weeks of incubation, most likely due to ammonia oxidisation, in which ammonium in artificial seawater (Table S1) is oxidised to nitrite and nitrate by nitrifying bacteria and archaea. In addition, negatively charged nitrites and nitrates can be adsorbed on Fe‐Mn concretions and released into solution when exposed to small pH changes at the start of incubation. We identified several nitrifiers across all samples (Figure 12), such as ammonia‐oxidising archaea *Candidatus Nitrosopumilus* (Qin et al. 2016) and ammonia‐oxidising bacteria *Nitrosomonas*, *Nitrospina* and *Nitrospira* (Koops and Pommerening‐Röser 2015; Sun et al. 2019; Daims et al. 2015), with the latter including species capable of complete nitrification (Daims et al. 2015) and was previously characterised in Baltic Sea Fe‐Mn concretions (Yli‐Hemminki et al. 2014; Reunamo et al. 2017). It should be noted that due to the addition of NaN_3_ into abiotic controls, N_2_O, NO_3_ and NO_2_ concentrations could not be determined, making comparisons between abiotic controls and biotic triplicates not possible. However, together with consistent geochemical and community composition data, received from biotic triplicates, we provide evidence of coexistence of several nitrifiers and denitrifies in Fe‐Mn concretions that have potential to contribute to the nitrogen cycle.

Phosphate decreased after 15 weeks of incubation, indicating its uptake by microbes and accumulation in their biomass (Figure 8). Alternatively, phosphate can also be adsorbed abiotically onto Fe(III) (hydr)oxides within concretions, while its abiotic adsorption on Mn (hydr)oxides likely plays a minor role (Anschutz et al. 2024). In the crust morphotype, P was adsorbed both biologically and abiotically, either onto concretions or the bottle walls (Figure 5; Figure 6), but precipitation on the bottle walls was enhanced in the presence of microbes. In discoidal and spheroidal morphotypes, P was released from the Fe‐Mn concretions but was later re‐adsorbed onto the bottle walls. The more columnar and porous microstructure of Mn‐rich discoidal and spheroidal concretions (Wasiljeff et al. 2024; Wasiljeff, Yu, et al. 2025) could promote greater habitat diversity, formation of anoxic microniches, and local hot spots for Fe and Mn reducers, releasing P when the binding surface is lost (Yli‐Hemminki et al. 2016). Abiotic dissolution of Fe‐Mn (hydr)oxides could also occur simultaneously. Bottle walls, however, are likely well‐oxygenated and thus favourable surfaces for formation of fresh Fe‐Mn (hydr)oxides, in which P can be re‐adsorbed.

## 5 Conclusions

Different concretion morphologies in the Gulf of Finland—crust, discoidal and spheroidal—host distinct bacterial and archaeal communities and metal accumulation patterns. Microbial activity enhanced metal precipitation most in spheroidal and discoidal morphotypes. Both morphotypes showed higher relative abundance of active Gammaproteobacteria, including several genera capable of Fe‐ and Mn‐oxidation, such as *Leptothrix*, *Gallionella*, and *Pseudomonas*, suggesting their role in enhanced metal accumulation. Furthermore, discoidal and spheroidal morphotypes had a higher relative abundance of active sulfate‐reducing bacteria, such as Sva0081 sediment group and SEEP‐SRB1 in class Desulfobacteria. Crust morphotype had a higher relative abundance of the genus *Candidatus Entotheonella*, and Mo was an important factor associated with crust communities, suggesting a potential role of *Entotheonella* sp. in the accumulation of Mo in crust concretion morphotype.

Despite revealed dissimilarities at the genus level, all three morphologies were dominated by bacteria, while the abundance of archaea remained small. The dominant bacterial phylum was Pseudomonadota, with the classes Alpha‐ and Gammaproteobacteria. In addition to genera with potential to oxidise metals, we identified several genera involved in Fe‐ and Mn reduction, such as *Rhodoferax* (Gammaproteobacteria) and *Acidiferrimicrobium* (Acidimicrobiia), highlighting their potential roles in metal cycling within Fe‐Mn concretions. Furthermore, we observed active genera participating in the nitrogen cycle and methane oxidation. The co‐occurrence of aerobic and anaerobic communities within the same Fe‐Mn concretion suggests microscale oxic‐suboxic‐anoxic zonation and redox heterogeneity, supporting diverse microbial populations in Fe‐Mn concretions.

The microbial community composition in Fe‐Mn concretions from the Gulf of Finland differs from that of the surrounding environment, suggesting that these concretions are unique microenvironments that serve as selective habitats for microbes in the Gulf of Finland. Importantly, each Fe‐Mn concretion morphotype hosts a characteristic microbial community with differing effects on many processes on the seafloor, such as metal enrichment and nutrient cycling.

## Author Contributions

**Lotta Purkamo:** supervision, conceptualization, methodology, project administration, writing – review and editing, investigation. **Renata Majamäki:** conceptualization, methodology, software, data curation, investigation, validation, formal analysis, visualization, writing – original draft, writing – review and editing, project administration, resources. **Pirjo Yli‐Hemminki:** conceptualization, methodology, writing – review and editing, supervision. **Joonas Wasiljeff:** conceptualization, methodology, validation, supervision, visualization, writing – review and editing, writing – original draft, formal analysis. **Lukas Kohl:** investigation, resources, formal analysis, writing – review and editing, validation. **Jenni Hultman:** supervision, conceptualization, methodology, project administration, resources, writing – review and editing. **Eero Asmala:** conceptualization, methodology, writing – review and editing, supervision. **Joonas J. Virtasalo:** supervision, funding acquisition, project administration, conceptualization, methodology, investigation, resources, writing – review and editing. **Johanna Muurinen:** conceptualization. **Kirsten S. Jørgensen:** conceptualization, methodology, writing – review and editing, supervision.

## Funding

This work was supported by the Research Council of Finland (332249) and the Foundation for Research of Natural Resources in Finland (20240020).

## Conflicts of Interest

The authors declare no conflicts of interest.

## Supporting information


**Table S1:** Composition of artificial brackish seawater of the Gulf of Finland. Fe and Mn concentrations were increased 30‐fold.
**Table S2:** Measured concentrations (ppb or μg/L) of trace elements and rare earth elements (REE) in artificial brackish seawater. REEs below detection limits are marked as < [concentration].
**Table S3:** Normalised methane CH_4_, carbon dioxide CO_2_, nitrous oxide N_2_O and oxygen O_2_ concentrations (ppm) from the headspace of microcosm bottles at week‐0 and week‐15 from site 1 (crust), site 2 (discoidal) and site 3 (spheroidal).
**Table S4:** Original element concentrations (μg/L) from the incubation solution of microcosms in site 1 (crust), site 2 (discoidal), and site 3 (spheroidal). A0‐C0 = biotic triplicates at 0‐week, A15–C15 = biotic triplicates at 15‐week, CTRL0 = abiotic control at 0‐week, CTRL15 = abiotic control at 15‐week.
**Table S5:** Normalised element concentrations (μg/L) from site 1 (crust), site 2 (discoidal), and site 3 (spheroidal). Normalised concentrations were calculated by dividing the original concentrations (Table S4) by measured concentrations of artificial seawater (Table S2). A0‐C0 = biotic triplicates at 0‐week, A15–C15 = biotic triplicates at 15‐week, CTRL0 = abiotic control at 0‐week, CTRL15 = abiotic control at 15‐week.
**Table S6:** Differences (μg/L) between the 0‐week and 15‐week incubation from site 1 (crust), site 2 (discoidal), and site 3 (spheroidal). The differences were calculated as relative changes using normalised concentrations (Table S5): the mean 0‐week value was subtracted from the mean 15‐week value, and divided by the mean 0‐week value.
**Table S7:** Original concentrations (μg/L) of the elements that were precipitated on bottle walls after 15 weeks of incubation from site 1 (crust), site 2 (discoidal), and site 3 (spheroidal). A‐15–C‐15 = biotic triplicates at 15‐week, CTRL‐15 = abiotic control at 15‐week.
**Table S8:** Original REE concentrations (ppb) from the incubation solution of microcosms in site 1 (crust), site 2 (discoidal), and site 3 (spheroidal). A0–C0 = biotic triplicates at 0‐week, A15‐C15 = biotic triplicates at 15‐week, CTRL0 = abiotic control at 0‐week, CTRL15 = abiotic control at 15‐week.
**Table S9:** REE concentrations from the incubation solution, normalised to Post‐Archean Australian Shale (PAAS). Normalised concentrations were calculated by dividing each (mean) original concentration by the corresponding PAAS value.
**Table S10:** Original REE concentrations (ppb) that were precipitated on bottle walls after 15 weeks of incubation from site 1 (crust), site 2 (discoidal), and site 3 (spheroidal). A‐15–C‐15 = biotic triplicates at 15‐week, CTRL‐15 = abiotic control at 15‐week.
**Table S11:** Normalised REE concentrations (ppb) that were precipitated on bottle walls after 15 weeks of incubation from site 1 (crust), site 2 (discoidal), and site 3 (spheroidal). Concentrations were normalised to Post‐Archean Australian Shale (PAAS) by dividing each (mean) original concentration by the corresponding PAAS value.
**Table S12:** Nitrate+nitrite, total nitrogen, and phosphate concentrations (μg/L) from the Artificial brackish seawater and from the incubation solution of microcosms in site 1 (crust), site 2 (discoidal), and site 3 (spheroidal). A‐15–C‐15 = biotic triplicates at 15‐week.
**Table S13A:** Shannon diversity index (*H*′) values from fluffy, sediment, and Fe‐Mn concretion samples collected immediately after sampling (intrinsic) in 2023. Samples are arranged based on collected environment.
**Table S13B:** Shannon diversity index (*H*′) values from fluffy, sediment, and Fe‐Mn concretion samples collected immediately after sampling (intrinsic) in 2022. Samples are arranged based on the collected environment.
**Table S14:** Shannon diversity index (*H*′) values from ‐Mn concretion samples collected from incubation experiments in 2023 and 2022. Samples are arranged based on the site.
**Table S15A:** ANOVA and Tukey HSD post hoc comparisons of Shannon diversity between environments from 2023 and 2022. Diff = difference in means, lwr and upr = confidence levels, adj. *p* = adjusted *p* values for all possible pairs.
**Table S15B:** ANOVA and Tukey HSD post hoc comparisons of Shannon diversity between morphotypes from incubation experiment in 2023. Welch's ANOVA, followed by Games–Howell post hoc comparisons were done from 2022 experiment. Diff = difference in means, lwr and upr = confidence levels, adj. *p* = adjusted *p* values for all possible pairs.
**Table S16:** PERMANOVA results based on Bray–Curtis distances from intrinsic fluffy, sediment, and Fe‐Mn concretion samples in 2023 and 2022. P_adj_BH = Benjamini–Hochberg.
**Table S17:** PERMANOVA results based on Bray‐Curtis distances from Fe‐Mn concretion samples collected from incubation experiments and envfit results from each element. Elements are arranged based on their adjusted *p* value. P_adj_BH = Benjamini–Hochberg.

## Data Availability

The data that support the findings of this study are openly available in NCBI Sequence Read Archive (SRA) at https://www.ncbi.nlm.nih.gov/sra, reference number PRJNA1417979.
