# Supplementary material for: Different Ferromanganese Concretion Morphologies Host Distinct Microbial Communities and Metal Accumulation Patterns in the Baltic Sea
Source: Environ Microbiol. 2026 Jul 23;28(7):e70390. doi: 10.1111/1462-2920.70390 (PMC13395527; doi:10.1111/1462-2920.70390)
Supplement: Supplementary file 1 — Table S1: Composition of artificial brackish seawater of the Gulf of Finland. Fe and Mn concentrations were increased 30‐fold. Table S2: Measured concentrations (ppb or μg/L) of trace elements and rare earth elements (REE) in artificial brackish seawater. REEs below detection limits are marked as < [concentration]. Table S3: Normalised methane CH4, carbon dioxide CO2, nitrous oxide N2O and oxygen O2 concentrations (ppm) from the headspace of microcosm bottles at week‐0 and week‐15 from site 1 (crust), site 2 (discoidal) and site 3 (spheroidal). Table S4: Original element concentrations (μg/L) from the incubation solution of microcosms in site 1 (crust), site 2 (discoidal), and site 3 (spheroidal). A0‐C0 = biotic triplicates at 0‐week, A15–C15 = biotic triplicates at 15‐week, CTRL0 = abiotic control at 0‐week, CTRL15 = abiotic control at 15‐week. Table S5: Normalised element concentrations (μg/L) from site 1 (crust), site 2 (discoidal), and site 3 (spheroidal). Normalised concentrations were calculated by dividing the original concentrations (Table S4) by measured concentrations of artificial seawater (Table S2). A0‐C0 = biotic triplicates at 0‐week, A15–C15 = biotic triplicates at 15‐week, CTRL0 = abiotic control at 0‐week, CTRL15 = abiotic control at 15‐week. Table S6: Differences (μg/L) between the 0‐week and 15‐week incubation from site 1 (crust), site 2 (discoidal), and site 3 (spheroidal). The differences were calculated as relative changes using normalised concentrations (Table S5): the mean 0‐week value was subtracted from the mean 15‐week value, and divided by the mean 0‐week value. Table S7: Original concentrations (μg/L) of the elements that were precipitated on bottle walls after 15 weeks of incubation from site 1 (crust), site 2 (discoidal), and site 3 (spheroidal). A‐15–C‐15 = biotic triplicates at 15‐week, CTRL‐15 = abiotic control at 15‐week. Table S8: Original REE concentrations (ppb) from the incubation solution of microcosms in site 1 (crust), site [file EMI-28-e70390-s001.docx]

Supplementary Material for

**Different ferromanganese concretion morphologies host distinct microbial communities and metal accumulation patterns in the Baltic Sea**

Renata Majamäki^1^*, Joonas Wasiljeff^1^, Lotta Purkamo^1^, Jenni Hultman^2,3^, Lukas Kohl^4,5^, Eero Asmala^1^, Pirjo Yli-Hemminki^2^, Kirsten S. Jørgensen^6^, Johanna Muurinen^3^ and Joonas J. Virtasalo^1^

^1^Geological Survey of Finland (GTK), Vuorimiehentie 5, 02151 Espoo, Finland

^2^Natural Resources Institute Finland (LUKE), Latokartanonkaari 9, 00790 Helsinki, Finland

^3^University of Helsinki, Department of Microbiology, Viikinkaari 9, 00790 Helsinki, Finland

^4^University of Helsinki, Department of Agricultural Sciences, Viikinkaari 9, 00790 Helsinki, Finland

^5^University of Eastern Finland, Department of Biological and Environmental Science, Yliopistonranta 8, 70210 Kuopio, Finland

^6^Finnish Environment Institute, Latokartanonkaari 11, 00790 Helsinki, Finland

*Corresponding author: Renata Majamäki ([renata.majamaki@gtk.fi](mailto:renata.majamaki@gtk.fi))

**Contents:**

Table S1 - S17

**Table S1.** Composition of artificial brackish seawater of the Gulf of Finland. Fe and Mn concentrations were increased 30-fold.

| **Macronutrients** | **Concentration mg/L** |
| --- | --- |
| NH_4_Cl | 16.13 |
| K_2_HPO_4_ | 1.55 |
| **Micronutrients** | **Concentration µg/L** |
| Na_2_WO_4_*2H_2_O | 0.165 |
| (NH_4_)_6_Mo_7_O_24_*2H_2_O | 0.44 |
| KBr | 0.6 |
| KI | 0.415 |
| ZnSO_4_*7H_2_O | 1.435 |
| Cd(NO_3_)_2_*4H_2_O | 0.77 |
| Co(NO_3_)_2_*6H_2_O | 0.73 |
| CuSO_4_*5H_2_O | 0.625 |
| NiSO_4_(NH_4_)_2_SO_4_*6H_2_O | 0.99 |
| Cr(NO_3_)_3_*9H_2_O | 0.205 |
| KAl(SO_4_)_2_*12H_2_O | 4.74 |
| V_2_O_5_ | 0.0445 |
| H_3_BO_3_ | 15.5 |
| FeCl_3_*6H_2_O | 4200 (Fe x 30 increase. original concentration 140 µg/L) |
| MnSO_4_*4H_2_O | 334.5 (Mn x 30 increase. original concentration 11.15 µg/L) |
| **Salts** | **Concentration g/L** |
| NaCl | 4.68 |
| MgCl_2_ | 1.36 |
| MgSO_4_*7H_2_O | 0.56 |
| CaCl_2_*2H_2_O | 0.46 |
| K_2_SO_4_ | 0.15 |
| CaCO_3_ | 0.02 |

**Table S2.** Measured concentrations (ppb or µg/L) of trace elements and rare earth elements (REE) in artificial brackish seawater. REEs below detection limits are marked as <[concentration].

| **Element** | **Concentration ppb (µg/L)** |
| --- | --- |
| P | 281.466 |
| Mn | 104.496 |
| Fe | 12.511 |
| Al | 77.516 |
| Co | 0.29 |
| Ni | 0.979 |
| V | 0.316 |
| Zn | 5.618 |
| Mo | 0.415 |
| Cr | 2.097 |
| Cu | 1.108 |
| Pb | 0.045 |
| Li | 1.214 |
|  |  |
| **Element** | **Concentration ppb (µg/L)** |
| La | 0.002 |
| Ce | 0.229 |
| Pr | 0.001 |
| Nd | <0.007 |
| Sm | <0.002 |
| Eu | <0.004 |
| Gd | <0.005 |
| Tb | <0.004 |
| Dy | <0.002 |
| Y | <0.113 |
| Ho | <0.019 |
| Er | <0.002 |
| Yb | <0.005 |
| Lu | <0.008 |

**Table S3.** Normalized methane CH_4_, carbon dioxide CO_2_, nitrous oxide N_2_O and oxygen O_2_ concentrations (ppm) from the headspace of microcosm bottles at week-0 and week-15 from site 1 (crust), site 2 (discoidal) and site 3 (spheroidal).

|  |  | Normalized headspace gas concentration (ppm) | | | |  |  | Normalized headspace gas concentration (ppm) | | | |
| --- | --- | --- | --- | --- | --- | --- | --- | --- | --- | --- | --- |
|  | Biotic triplicates  (0-week) | CH_4_ | CO_2_ | N_2_O | O_2_ |  | Biotic triplicates  (15-week) | CH_4_ | CO_2_ | N_2_O | O_2_ |
| **Site 1** | 1 A0 | 2.45 | 1237 | 1.01 | 210011 |  | 1 A15 | 0.66 | 4061 | 2.59 | 180909 |
| **(crust)** | 1 B0 | 2.32 | 906 | 0.89 | 209643 |  | 1 B15 | 0.85 | 9125 | 0.69 | 127831 |
|  | 1 C0 | 2.26 | 1077 | 0.59 | 208752 |  | 1 C15 | 0.68 | 5318 | 22.12 | 167624 |
|  | Mean (std) | 2.34 (± 0.08) | 1073 (± 135) | 0.83 (± 0.18) | 209469 (± 529) |  | Mean (std) | 0.73 (± 0.08) | 6168 (± 2153) | 8.47 (± 9.69) | 158788 (± 22552) |
|  | 1 abiotic control 0 | 2.35 | 746 | 5.68 | 211847 |  | 1 abiotic control 15 | 2.32 | 1750 | 51.28 | 209683 |
|  |  |  |  |  |  |  |  |  |  |  |  |
| **Site 2** | 2 A0 | 2.21 | 1267 | 1.21 | 208856 |  | 2 A15 | 0.76 | 14049 | 2.13 | 174390 |
| **(discoidal)** | 2 B0 | 2.32 | 886 | 1.02 | 210459 |  | 2 B15 | 0.64 | 6613 | 1.53 | 174714 |
|  | 2 C0 | 2.29 | 1289 | 1.40 | 206568 |  | 2 C15 | 0.77 | 5856 | 1.55 | 180040 |
|  | Mean (std) | 2.27 (± 0.04) | 1147 (± 185) | 1.21 (±0.16) | 208628 (± 1596) |  | Mean (std) | 0.73 (±0.06) | 8839 (± 3696) | 1.74 (± 0.28) | 176382 (± 2590) |
|  | 2 abiotic control 0 | 2.23 | 853 | 14.72 | 212246 |  | 2 abiotic control 15 | 2.31 | 1776 | 50.07 | 209999 |
|  |  |  |  |  |  |  |  |  |  |  |  |
| **Site 3** | 3 A0 | 2.33 | 1082 | 0.91 | 207959 |  | 3 A15 | 1.21 | 21009 | 1.61 | 187317 |
| **(spheroidal)** | 3 B0 | 2.28 | 1224 | 1.07 | 209665 |  | 3 B15 | 2.09 | 5515 | 1.21 | 178808 |
|  | 3 C0 | 2.18 | *37328** | 0.98 | 201986 |  | 3 C15 | 1.07 | 5937 | 1.55 | 179617 |
|  | Mean (std) | 2.26 (± 0.06) | 1153 (± 71) | 0.99 (± 0.07) | 206537 (± 3292) |  | Mean (std) | 1.45 (± 0.45) | 10821 (± 7207) | 1.46 (±0.17) | 181914 (± 3834) |
|  | 3 abiotic control 0 | 2.27 | 790 | 8.42 | 212329 |  | 3 abiotic control 15 | 2.38 | 2120 | 50.62 | 209540 |
|  |  |  |  |  |  |  |  |  |  |  |  |

**not included in the standard deviation*

**Table S4.** Original element concentrations (µg/L) from the incubation solution of microcosms in site 1 (crust), site 2 (discoidal), and site 3 (spheroidal). A0-C0 = biotic triplicates at 0-week, A15-C15 = biotic triplicates at 15-week, CTRL0 = abiotic control at 0-week, CTRL15 = abiotic control at 15-week.

|  |  | Al | P | V | Cr | Mn | Fe | Co | Ni | Cu | Zn | Mo | Pb | Li |
| --- | --- | --- | --- | --- | --- | --- | --- | --- | --- | --- | --- | --- | --- | --- |
| **Site 1 (crust)** | 1A0 | 4.09 | 81.74 | 0.41 | 1.88 | 34.78 | 11.95 | 0.25 | 1.17 | 1.22 | 8.14 | 0.63 | 1.95 | 21.49 |
|  | 1B0 | 2.96 | 86.25 | 0.25 | 1.86 | 2.82 | 11.54 | 0.17 | 0.96 | 0.88 | 4.92 | 0.74 | 0.38 | 29.01 |
|  | 1C0 | 3.67 | 123.68 | 0.52 | 1.84 | 54.45 | 11.95 | 0.24 | 1.42 | 1.54 | 11.80 | 0.92 | 0.38 | 9.20 |
|  | 1A15 | 2.98 | 77.03 | 0.16 | 0.99 | 4.75 | 26.98 | 0.19 | 1.15 | 6.47 | 29.91 | 0.46 | 0.08 | 37.54 |
|  | 1B15 | 2.14 | 114.69 | 0.40 | 0.93 | 0.75 | 33.82 | 1.26 | 5.87 | 12.50 | 13.98 | 0.21 | 0.07 | 16.01 |
|  | 1C15 | 9.85 | 50.73 | 0.19 | 0.90 | 0.29 | 38.32 | 0.42 | 2.08 | 2.32 | 5.57 | 0.42 | 0.04 | 40.32 |
|  | 1CTRL0 | 5.77 | 177.18 | 2.18 | 1.90 | 915.41 | 15.09 | 0.50 | 1.69 | 2.14 | 6.02 | 2.25 | 0.36 | 18.15 |
|  | 1CTRL15 | 2.68 | 146.12 | 0.24 | 0.91 | 12.13 | 28.16 | 0.94 | 1.51 | 10.79 | 12.46 | 1.30 | 0.05 | 50.96 |
|  |  |  |  |  |  |  |  |  |  |  |  |  |  |  |
| **Site 2 (discoidal)** | 2A0 | 4.63 | 80.04 | 0.18 | 1.85 | 18.17 | 14.32 | 0.24 | 1.01 | 1.56 | 14.64 | 0.30 | 0.16 | 19.98 |
|  | 2B0 | 2.98 | 86.33 | 0.19 | 1.88 | 15.25 | 12.70 | 0.20 | 0.91 | 1.47 | 10.23 | 0.34 | 0.15 | 12.22 |
|  | 2C0 | 4.81 | 71.34 | 0.17 | 1.87 | 8.95 | 13.20 | 0.19 | 0.95 | 1.70 | 12.98 | 0.31 | 0.11 | 20.02 |
|  | 2A15 | 6.26 | 152.47 | 0.24 | 0.95 | 0.83 | 52.09 | 0.40 | 0.92 | 3.72 | 26.05 | 0.17 | 0.04 | 27.17 |
|  | 2B15 | 2.93 | 91.13 | 0.18 | 1.03 | 0.36 | 44.30 | 0.17 | 1.13 | 1.61 | 6.76 | 0.16 | 0.01 | 33.16 |
|  | 2C15 | 6.16 | 99.37 | 0.22 | 0.97 | 0.57 | 47.02 | 0.20 | 0.90 | 2.14 | 14.32 | 0.29 | 0.03 | 34.14 |
|  | 2CTRL0 | 3.82 | 132.03 | 0.26 | 1.94 | 97.61 | 13.95 | 0.25 | 1.15 | 3.19 | 12.92 | 0.95 | 0.12 | 20.06 |
|  | 2CTRL15 | 21.93 | 188.48 | 0.32 | 0.91 | 117.11 | 42.91 | 1.22 | 1.82 | 9.19 | 45.45 | 1.81 | 0.01 | 31.10 |
|  |  |  |  |  |  |  |  |  |  |  |  |  |  |  |
| **Site 3 (spheroidal)** | 3A0 | 6.28 | 139.76 | 1.17 | 1.90 | 900.28 | 19.48 | 0.70 | 1.56 | 1.91 | 13.58 | 0.82 | 0.37 | 5.53 |
|  | 3B0 | 2.90 | 114.51 | 0.29 | 1.91 | 179.49 | 15.18 | 0.43 | 1.11 | 1.72 | 13.03 | 0.27 | 0.25 | 11.47 |
|  | 3C0 | 5.36 | 263.62 | 0.55 | 1.88 | 1762.20 | 39.41 | 1.35 | 2.17 | 2.55 | 36.46 | 0.36 | 0.38 | 9.38 |
|  | 3A15 | 1.80 | 168.17 | 0.22 | 1.01 | 1.99 | 65.59 | 1.55 | 0.96 | 2.88 | 28.31 | 0.15 | 0.04 | 23.86 |
|  | 3B15 | 5.59 | 192.52 | 0.33 | 0.99 | 0.84 | 52.64 | 0.76 | 1.03 | 4.32 | 16.95 | 0.29 | 0.05 | 12.49 |
|  | 3C15 | 3.45 | 164.37 | 0.27 | 1.01 | 1.09 | 49.48 | 0.66 | 1.05 | 3.82 | 22.02 | 0.29 | 0.02 | 22.31 |
|  | 3CTRL0 | 2.41 | 159.78 | 0.45 | 1.94 | 708.16 | 14.98 | 0.53 | 1.43 | 3.23 | 10.72 | 1.83 | 0.27 | 10.53 |
|  | 3CTRL15 | 2.39 | 231.05 | 0.35 | 0.89 | 47.65 | 29.62 | 0.95 | 1.37 | 9.45 | 60.65 | 1.98 | 0.04 | 40.65 |

**Table S5.** Normalised element concentrations (µg/L) from site 1 (crust), site 2 (discoidal), and site 3 (spheroidal). Normalised concentrations were calculated by dividing the original concentrations (Table S4) by measured concentrations of artificial seawater (Table S2). A0-C0 = biotic triplicates at 0-week, A15-C15 = biotic triplicates at 15-week, CTRL0 = abiotic control at 0-week, CTRL15 = abiotic control at 15-week.

|  |  | *µg/L* | *µg/L* | *µg/L* | *µg/L* | *µg/L* | *µg/L* | *µg/L* | *µg/L* | *µg/L* | *µg/L* | *µg/L* | *µg/L* | *µg/L* |
| --- | --- | --- | --- | --- | --- | --- | --- | --- | --- | --- | --- | --- | --- | --- |
| **Site 1 (crust)** |  | Al | P | V | Cr | Mn | Fe | Co | Ni | Cu | Zn | Mo | Pb | Li |
|  | 1A0 | 0.053 | 0.290 | 1.285 | 0.898 | 0.333 | 0.955 | 0.845 | 1.199 | 1.098 | 1.449 | 1.520 | 43.422 | 17.705 |
|  | 1B0 | 0.038 | 0.306 | 0.782 | 0.888 | 0.027 | 0.922 | 0.597 | 0.978 | 0.794 | 0.876 | 1.788 | 8.489 | 23.894 |
|  | 1C0 | 0.047 | 0.439 | 1.639 | 0.878 | 0.521 | 0.955 | 0.824 | 1.450 | 1.387 | 2.100 | 2.224 | 8.356 | 7.579 |
|  | Mean (0-week) | 0.046 | 0.345 | 1.235 | 0.888 | 0.294 | 0.944 | 0.755 | 1.209 | 1.093 | 1.475 | 1.844 | 20.089 | 16.393 |
|  | std (0-week) | 0.006 | 0.067 | 0.352 | 0.008 | 0.204 | 0.015 | 0.112 | 0.193 | 0.242 | 0.500 | 0.290 | 16.499 | 6.725 |
|  | 1A15 | 0.038 | 0.274 | 0.519 | 0.473 | 0.045 | 2.157 | 0.662 | 1.178 | 5.840 | 5.325 | 1.106 | 1.867 | 30.923 |
|  | 1B15 | 0.028 | 0.407 | 1.272 | 0.443 | 0.007 | 2.703 | 4.328 | 5.996 | 11.277 | 2.489 | 0.499 | 1.600 | 13.187 |
|  | 1C15 | 0.127 | 0.180 | 0.611 | 0.429 | 0.003 | 3.063 | 1.438 | 2.121 | 2.097 | 0.991 | 1.002 | 0.800 | 33.214 |
|  | Mean (15-week) | 0.064 | 0.287 | 0.801 | 0.448 | 0.018 | 2.641 | 2.143 | 3.098 | 6.405 | 2.935 | 0.869 | 1.422 | 25.775 |
|  | std (15-week) | 0.045 | 0.093 | 0.336 | 0.018 | 0.019 | 0.373 | 1.577 | 2.085 | 3.769 | 1.797 | 0.265 | 0.453 | 8.950 |
|  | 1CTRL0 | 0.074 | 0.630 | 6.889 | 0.906 | 8.760 | 1.206 | 1.734 | 1.730 | 1.931 | 1.072 | 5.419 | 8.067 | 14.946 |
|  | 1CTRL15 | 0.035 | 0.519 | 0.759 | 0.432 | 0.116 | 2.251 | 3.228 | 1.546 | 9.742 | 2.217 | 3.135 | 1.133 | 41.974 |
|  |  |  |  |  |  |  |  |  |  |  |  |  |  |  |
| **Site 2 (discoidal)** |  | Al | P | V | Cr | Mn | Fe | Co | Ni | Cu | Zn | Mo | Pb | Li |
|  | 2A0 | 0.060 | 0.284 | 0.579 | 0.882 | 0.174 | 1.144 | 0.828 | 1.035 | 1.403 | 2.606 | 0.720 | 3.622 | 16.457 |
|  | 2B0 | 0.038 | 0.307 | 0.611 | 0.895 | 0.146 | 1.015 | 0.679 | 0.928 | 1.323 | 1.820 | 0.812 | 3.267 | 10.063 |
|  | 2C0 | 0.062 | 0.253 | 0.528 | 0.890 | 0.086 | 1.055 | 0.669 | 0.968 | 1.534 | 2.311 | 0.745 | 2.444 | 16.488 |
|  | Mean (0-week) | 0.053 | 0.282 | 0.573 | 0.889 | 0.135 | 1.072 | 0.725 | 0.977 | 1.420 | 2.246 | 0.759 | 3.111 | 14.336 |
|  | std (0-week) | 0.011 | 0.022 | 0.034 | 0.005 | 0.037 | 0.054 | 0.072 | 0.044 | 0.087 | 0.324 | 0.039 | 0.493 | 3.021 |
|  | 2A15 | 0.081 | 0.542 | 0.763 | 0.454 | 0.008 | 4.164 | 1.386 | 0.936 | 3.360 | 4.636 | 0.405 | 0.778 | 22.378 |
|  | 2B15 | 0.038 | 0.324 | 0.570 | 0.493 | 0.003 | 3.541 | 0.600 | 1.153 | 1.456 | 1.203 | 0.393 | 0.289 | 27.313 |
|  | 2C15 | 0.079 | 0.353 | 0.680 | 0.462 | 0.005 | 3.759 | 0.686 | 0.919 | 1.929 | 2.549 | 0.687 | 0.600 | 28.119 |
|  | Mean (15-week) | 0.066 | 0.406 | 0.671 | 0.470 | 0.006 | 3.821 | 0.891 | 1.003 | 2.248 | 2.796 | 0.495 | 0.556 | 25.937 |
|  | std (15-week) | 0.020 | 0.097 | 0.079 | 0.016 | 0.002 | 0.258 | 0.352 | 0.107 | 0.810 | 1.413 | 0.136 | 0.202 | 2.538 |
|  | 2CTRL0 | 0.049 | 0.469 | 0.820 | 0.924 | 0.934 | 1.115 | 0.855 | 1.172 | 2.879 | 2.300 | 2.292 | 2.644 | 16.521 |
|  | 2CTRL15 | 0.283 | 0.670 | 1.000 | 0.436 | 1.121 | 3.430 | 4.193 | 1.855 | 8.294 | 8.090 | 4.352 | 0.311 | 25.619 |

| **Site 3 (spheroidal)** |  | Al | P | V | Cr | Mn | Fe | Co | Ni | Cu | Zn | Mo | Pb | Li |
| --- | --- | --- | --- | --- | --- | --- | --- | --- | --- | --- | --- | --- | --- | --- |
|  | 3A0 | 0.081 | 0.497 | 3.703 | 0.905 | 8.615 | 1.557 | 2.417 | 1.592 | 1.719 | 2.417 | 1.966 | 8.311 | 4.554 |
|  | 3B0 | 0.037 | 0.407 | 0.918 | 0.910 | 1.718 | 1.213 | 1.497 | 1.130 | 1.550 | 2.319 | 0.658 | 5.444 | 9.446 |
|  | 3C0 | 0.069 | 0.937 | 1.744 | 0.896 | 16.864 | 3.150 | 4.652 | 2.219 | 2.300 | 6.489 | 0.875 | 8.333 | 7.724 |
|  | Mean (0-week) | 0.062 | 0.613 | 2.121 | 0.904 | 9.066 | 1.973 | 2.855 | 1.647 | 1.856 | 3.742 | 1.166 | 7.363 | 7.242 |
|  | std (0-week) | 0.018 | 0.232 | 1.168 | 0.006 | 6.192 | 0.844 | 1.325 | 0.446 | 0.321 | 1.943 | 0.573 | 1.357 | 2.026 |
|  | 3A15 | 0.023 | 0.597 | 0.684 | 0.483 | 0.019 | 5.243 | 5.359 | 0.981 | 2.603 | 5.039 | 0.352 | 0.778 | 19.652 |
|  | 3B15 | 0.072 | 0.684 | 1.047 | 0.473 | 0.008 | 4.208 | 2.624 | 1.053 | 3.895 | 3.017 | 0.694 | 1.089 | 10.289 |
|  | 3C15 | 0.044 | 0.584 | 0.842 | 0.481 | 0.010 | 3.955 | 2.283 | 1.069 | 3.449 | 3.919 | 0.704 | 0.511 | 18.378 |
|  | Mean (15-week) | 0.047 | 0.622 | 0.858 | 0.479 | 0.012 | 4.468 | 3.422 | 1.034 | 3.316 | 3.992 | 0.583 | 0.793 | 16.107 |
|  | std (15-week) | 0.020 | 0.044 | 0.149 | 0.004 | 0.005 | 0.557 | 1.377 | 0.039 | 0.536 | 0.827 | 0.164 | 0.236 | 4.146 |
|  | 3CTRL0 | 0.031 | 0.568 | 1.434 | 0.923 | 6.777 | 1.197 | 1.814 | 1.461 | 2.915 | 1.909 | 4.405 | 6.067 | 8.670 |
|  | 3CTRL15 | 0.031 | 0.821 | 1.120 | 0.425 | 0.456 | 2.367 | 3.279 | 1.403 | 8.531 | 10.796 | 4.764 | 0.956 | 33.485 |

**Table S6.** Differences (µg/L) between the 0-week and 15-week incubation from site 1 (crust), site 2 (discoidal), and site 3 (spheroidal). The differences were calculated as relative changes using normalised concentrations (Table S5): the mean 0-week value was subtracted from the mean 15-week value, and divided by the mean 0-week value.

|  |  | *µg/L* | *µg/L* | *µg/L* | *µg/L* | *µg/L* | *µg/L* | *µg/L* | *µg/L* | *µg/L* | *µg/L* | *µg/L* | *µg/L* | *µg/L* |
| --- | --- | --- | --- | --- | --- | --- | --- | --- | --- | --- | --- | --- | --- | --- |
|  |  | Al | P | V | Cr | Mn | Fe | Co | Ni | Cu | Zn | Mo | Pb | Li |
| **Site 1 (crust)** | Biotic triplicates | 0.396 | -0.169 | -0.352 | -0.495 | -0.937 | 1.797 | 1.837 | 1.562 | 4.859 | 0.990 | -0.529 | -0.929 | 0.572 |
|  | Abiotic control | -0.536 | -0.175 | -0.890 | -0.524 | -0.987 | 0.866 | 0.861 | -0.106 | 4.046 | 1.068 | -0.422 | -0.860 | 1.808 |
|  | std (biotic triplicates) | 0.025 | 0.080 | 0.344 | 0.013 | 0.111 | 0.194 | 0.845 | 1.139 | 2.005 | 1.148 | 0.278 | 8.476 | 7.837 |
|  |  |  |  |  |  |  |  |  |  |  |  |  |  |  |
|  |  | Al | P | V | Cr | Mn | Fe | Co | Ni | Cu | Zn | Mo | Pb | Li |
| **Site 2 (discoidal)** | Biotic triplicates | 0.236 | 0.443 | 0.171 | -0.472 | -0.959 | 2.566 | 0.228 | 0.026 | 0.583 | 0.245 | -0.348 | -0.821 | 0.809 |
|  | Abiotic control | 4.740 | 0.428 | 0.220 | -0.528 | 0.200 | 2.076 | 3.903 | 0.583 | 1.881 | 2.518 | 0.899 | -0.882 | 0.551 |
|  | std (biotic triplicates) | 0.015 | 0.059 | 0.056 | 0.011 | 0.019 | 0.156 | 0.212 | 0.075 | 0.448 | 0.868 | 0.087 | 0.348 | 2.780 |
|  |  |  |  |  |  |  |  |  |  |  |  |  |  |  |
|  |  | Al | P | V | Cr | Mn | Fe | Co | Ni | Cu | Zn | Mo | Pb | Li |
| **Site 3 (spheroidal)** | Biotic triplicates | -0.254 | 0.014 | -0.596 | -0.470 | -0.999 | 1.265 | 0.198 | -0.372 | 0.786 | 0.067 | -0.500 | -0.892 | 1.224 |
|  | Abiotic control | -0.005 | 0.446 | -0.219 | -0.540 | -0.933 | 0.977 | 0.808 | -0.039 | 1.926 | 4.656 | 0.082 | -0.842 | 2.862 |
|  | std (biotic triplicates) | 0.019 | 0.138 | 0.658 | 0.005 | 3.098 | 0.700 | 1.351 | 0.242 | 0.429 | 1.385 | 0.368 | 0.796 | 3.086 |

**Table S7.** Original concentrations (µg/L) of the elements that were precipitated on bottle walls after 15 weeks of incubation from site 1 (crust), site 2 (discoidal), and site 3 (spheroidal). A-15 – C-15 = biotic triplicates at 15-week, CTRL-15 =abiotic control at 15-week.

|  |  | *µg/L* | *µg/L* | *µg/L* | *µg/L* | *µg/L* | *µg/L* | *µg/L* | *µg/L* | *µg/L* | *µg/L* | *µg/L* | *µg/L* |  |
| --- | --- | --- | --- | --- | --- | --- | --- | --- | --- | --- | --- | --- | --- | --- |
| **Site 1 (crust)** |  | Al | P | V | Cr | Mn | Fe | Co | Ni | Cu | Zn | Mo | Pb |  |
|  | 1A-15 | 1527.05 | 213.39 | 2.09 | 3.18 | 236.42 | 1474.98 | 0.59 | 1.28 | 2.84 | 6.94 | 0.172 | 2.298 |  |
|  | 1B-15 | 1706.07 | 254.45 | 3.55 | 3.64 | 568.87 | 2690.02 | 1.41 | 3.69 | 3.19 | 8.93 | 0.377 | 2.876 |  |
|  | 1C-15 | 2273.95 | 228.66 | 4.47 | 5.40 | 705.04 | 3465.78 | 1.81 | 3.69 | 4.43 | 9.75 | 0.497 | 1.915 |  |
|  | Mean (biotic triplicates) | 1835.69 | 232.16 | 3.37 | 4.07 | 503.45 | 2543.59 | 1.27 | 2.89 | 3.49 | 8.54 | 0.35 | 2.36 |  |
|  | Std (biotic triplicates) | 318.40 | 16.94 | 0.98 | 0.96 | 196.83 | 819.31 | 0.51 | 1.14 | 0.68 | 1.18 | 0.13 | 0.40 |  |
|  | 1CTRL-15 | 674.68 | 96.21 | 0.91 | 2.63 | 185.98 | 893.67 | 0.31 | 0.72 | 2.33 | 4.36 | 0.135 | 0.882 |  |
|  |  |  |  |  |  |  |  |  |  |  |  |  |  |  |
| **Site 2 (discoidal)** |  | Al | P | V | Cr | Mn | Fe | Co | Ni | Cu | Zn | Mo | Pb |  |
|  | 2A-15 | 600.04 | 165.66 | 0.79 | 2.70 | 141.83 | 782.90 | 0.30 | 1.43 | 2.00 | 4.94 | 0.065 | 1.918 |  |
|  | 2B-15 | 524.21 | 145.19 | 0.91 | 2.63 | 155.50 | 1303.92 | 0.32 | 1.11 | 1.10 | 3.90 | 0.143 | 0.934 |  |
|  | 2C-15 | 893.21 | 268.06 | 1.60 | 2.38 | 71.13 | 984.86 | 0.34 | 0.76 | 1.20 | 4.80 | 0.035 | 4.846 |  |
|  | Mean (biotic triplicates) | 672.49 | 192.97 | 1.10 | 2.57 | 122.82 | 1023.89 | 0.32 | 1.10 | 1.43 | 4.55 | 0.08 | 2.57 |  |
|  | Std (biotic triplicates) | 159.11 | 53.75 | 0.36 | 0.14 | 36.97 | 214.49 | 0.02 | 0.27 | 0.40 | 0.46 | 0.05 | 1.66 |  |
|  | 2CTRL-15 | 687.63 | 113.84 | 1.96 | 2.71 | 659.64 | 1389.87 | 0.55 | 2.08 | 1.90 | 6.89 | 0.36 | 1.226 |  |
|  |  |  |  |  |  |  |  |  |  |  |  |  |  |  |
| **Site 3 (spheroidal)** |  | Al | P | V | Cr | Mn | Fe | Co | Ni | Cu | Zn | Mo | Pb |  |
|  | 3A-15 | 753.10 | 164.47 | 1.11 | 2.60 | 220.09 | 1273.59 | 0.49 | 0.99 | 1.36 | 4.55 | 0.113 | 1.395 |  |
|  | 3B-15 | 1339.72 | 323.93 | 4.53 | 3.72 | 2589.66 | 4046.24 | 1.79 | 2.53 | 2.42 | 14.01 | 1.061 | 2.299 |  |
|  | 3C-15 | 1005.83 | 203.29 | 1.49 | 3.06 | 618.58 | 1555.54 | 0.82 | 1.12 | 1.42 | 5.26 | 0.294 | 3.324 |  |
|  | Mean (biotic triplicates) | 1032.88 | 230.56 | 2.38 | 3.13 | 1142.77 | 2291.79 | 1.03 | 1.55 | 1.73 | 7.94 | 0.49 | 2.34 |  |
|  | Std (biotic triplicates) | 240.25 | 67.89 | 1.53 | 0.46 | 1035.96 | 1245.91 | 0.55 | 0.69 | 0.49 | 4.30 | 0.41 | 0.79 |  |
|  | 3 CTRL-15 | 750.74 | 184.81 | 1.25 | 2.15 | 184.96 | 1901.66 | 0.50 | 0.79 | 1.51 | 5.03 | 0.265 | 1.284 |  |

**Table S8.** Original REE concentrations (ppb) from the incubation solution of microcosms in site 1 (crust), site 2 (discoidal), and site 3 (spheroidal). A0-C0 = biotic triplicates at 0-week, A15-C15 = biotic triplicates at 15-week, CTRL0 = abiotic control at 0-week, CTRL15 = abiotic control at 15-week.

|  |  |  | | | |  | | | | |  |  | | | |
| --- | --- | --- | --- | --- | --- | --- | --- | --- | --- | --- | --- | --- | --- | --- | --- |
|  |  | LREE | | | | MREE | | | | |  | HREE | | | |
|  |  | **La** | **Ce** | **Pr** | **Nd** | **Sm** | **Eu** | **Gd** | **Tb** | **Dy** | **Y** | **Ho** | **Er** | **Yb** | **Lu** |
| **Site 1 - crust** | 1A0 | 0.036 | 0.244 | 0.016 | 0.073 | 0.025 | 0.009 | 0.025 | 0.009 | 0.025 | 0.234 | <0.019 | 0.02 | 0.014 | <0.008 |
|  | 1B0 | 0.022 | 0.232 | 0.009 | 0.045 | 0.016 | 0.005 | 0.021 | <0.004 | 0.018 | 0.183 | <0.019 | 0.014 | 0.011 | <0.008 |
|  | 1C0 | 0.039 | 0.247 | 0.017 | 0.086 | 0.027 | 0.007 | 0.03 | 0.005 | 0.031 | 0.245 | <0.019 | 0.017 | 0.014 | <0.008 |
|  | Mean (0-week) | 0.032 | 0.241 | 0.014 | 0.068 | 0.023 | 0.007 | 0.025 | 0.007 | 0.025 | 0.221 |  | 0.017 | 0.013 |  |
|  | 1A15 | 0.009 | 0.011 | 0.003 | 0.018 | 0.007 | 0.004 | 0.007 | <0.004 | 0.02 | 0.282 | <0.019 | 0.073 | 0.158 | 0.034 |
|  | 1B15 | 0.02 | 0.009 | 0.006 | 0.031 | 0.012 | 0.007 | 0.012 | 0.005 | 0.041 | 0.459 | <0.019 | 0.107 | 0.244 | 0.051 |
|  | 1C15 | 0.019 | 0.027 | 0.006 | 0.018 | 0.006 | 0.004 | <0.005 | <0.004 | 0.013 | 0.142 | <0.019 | 0.034 | 0.109 | 0.027 |
|  | Mean (15-week) | 0.016 | 0.016 | 0.005 | 0.022 | 0.008 | 0.005 | 0.010 | 0.005 | 0.025 | 0.294 |  | 0.071 | 0.170 | 0.037 |
|  | 1CTRL0 | 0.023 | 0.256 | 0.009 | 0.051 | 0.014 | 0.005 | 0.014 | <0.004 | 0.014 | 0.152 | <0.019 | 0.010 | 0.007 | <0.008 |
|  | 1CTRL15 | 0.012 | 0.017 | 0.004 | 0.024 | 0.009 | <0.004 | 0.011 | <0.004 | 0.045 | 0.616 | 0.019 | 0.133 | 0.254 | 0.049 |
|  |  |  |  |  |  |  |  |  |  |  |  |  |  |  |  |
| **Site 2 - discoidal** |  | **La** | **Ce** | **Pr** | **Nd** | **Sm** | **Eu** | **Gd** | **Tb** | **Dy** | **Y** | **Ho** | **Er** | **Yb** | **Lu** |
|  | 2A0 | 0.066 | 0.272 | 0.025 | 0.143 | 0.037 | 0.007 | 0.044 | 0.006 | 0.044 | 0.367 | <0.019 | 0.026 | 0.019 | <0.008 |
|  | 2B0 | 0.066 | 0.259 | 0.026 | 0.14 | 0.032 | 0.009 | 0.038 | 0.007 | 0.037 | 0.324 | <0.019 | 0.025 | 0.015 | <0.008 |
|  | 2C0 | 0.062 | 0.255 | 0.024 | 0.133 | 0.04 | 0.009 | 0.051 | 0.007 | 0.06 | 0.527 | <0.019 | 0.037 | 0.024 | <0.008 |
|  | Mean (0-week) | 0.065 | 0.262 | 0.025 | 0.139 | 0.036 | 0.008 | 0.044 | 0.007 | 0.047 | 0.406 |  | 0.029 | 0.019 |  |
|  | 2A15 | 0.024 | 0.015 | 0.007 | 0.039 | 0.011 | 0.005 | 0.017 | 0.005 | 0.067 | 0.772 | 0.032 | 0.202 | 0.314 | 0.049 |
|  | 2B15 | 0.012 | 0.01 | 0.003 | 0.019 | 0.003 | <0.004 | 0.006 | <0.004 | 0.023 | 0.322 | <0.019 | 0.094 | 0.188 | 0.032 |
|  | 2C15 | 0.019 | 0.018 | 0.007 | 0.037 | 0.015 | 0.005 | 0.022 | 0.006 | 0.073 | 0.948 | 0.032 | 0.174 | 0.231 | 0.039 |
|  | Mean (15-week) | 0.018 | 0.014 | 0.006 | 0.032 | 0.010 | 0.005 | 0.015 | 0.006 | 0.054 | 0.681 | 0.032 | 0.157 | 0.244 | 0.040 |
|  | 2CTRL0 | 0.047 | 0.307 | 0.021 | 0.114 | 0.031 | 0.007 | 0.035 | 0.006 | 0.039 | 0.429 | <0.019 | 0.026 | 0.018 | <0.008 |
|  | 2CTRL15 | 0.029 | 0.046 | 0.008 | 0.042 | 0.015 | 0.005 | 0.024 | 0.005 | 0.083 | 1.011 | 0.036 | 0.178 | 0.180 | 0.025 |

|  |  | LREE | | | | MREE | | | | |  | HREE | | | |
| --- | --- | --- | --- | --- | --- | --- | --- | --- | --- | --- | --- | --- | --- | --- | --- |
| **Site 3 - spheroidal** |  | **La** | **Ce** | **Pr** | **Nd** | **Sm** | **Eu** | **Gd** | **Tb** | **Dy** | **Y** | **Ho** | **Er** | **Yb** | **Lu** |
|  | 3A0 | 0.045 | 0.311 | 0.015 | 0.101 | 0.026 | 0.008 | 0.035 | 0.005 | 0.023 | 0.213 | <0.019 | 0.014 | 0.009 | <0.008 |
|  | 3B0 | 0.033 | 0.267 | 0.014 | 0.086 | 0.026 | 0.007 | 0.03 | 0.005 | 0.03 | 0.317 | <0.019 | 0.016 | 0.011 | <0.008 |
|  | 3C0 | 0.046 | 0.285 | 0.02 | 0.102 | 0.039 | 0.017 | 0.039 | 0.014 | 0.057 | 0.456 | <0.019 | 0.038 | 0.031 | <0.008 |
|  | Mean (0-week) | 0.041 | 0.288 | 0.016 | 0.096 | 0.030 | 0.011 | 0.035 | 0.008 | 0.037 | 0.329 |  | 0.023 | 0.017 |  |
|  | 3A15 | 0.014 | 0.014 | 0.004 | 0.024 | 0.01 | <0.004 | 0.013 | <0.004 | 0.044 | 0.597 | 0.023 | 0.143 | 0.189 | 0.031 |
|  | 3B15 | 0.032 | 0.037 | 0.011 | 0.074 | 0.024 | 0.007 | 0.038 | 0.009 | 0.124 | 1.375 | 0.051 | 0.222 | 0.243 | 0.033 |
|  | 3C15 | 0.017 | 0.014 | 0.006 | 0.039 | 0.014 | 0.004 | 0.022 | 0.006 | 0.093 | 1.183 | 0.043 | 0.22 | 0.242 | 0.037 |
|  | Mean (15-week) | 0.021 | 0.022 | 0.007 | 0.046 | 0.016 | 0.006 | 0.024 | 0.008 | 0.087 | 1.052 | 0.039 | 0.195 | 0.225 | 0.034 |
|  | 3CTRL0 | 0.019 | 0.259 | 0.007 | 0.045 | 0.012 | 0.004 | 0.015 | <0.004 | 0.016 | 0.166 | <0.019 | 0.008 | 0.006 | <0.008 |
|  | 3CTRL15 | 0.014 | 0.021 | 0.007 | 0.042 | 0.012 | 0.004 | 0.026 | 0.006 | 0.084 | 1.162 | 0.031 | 0.153 | 0.174 | 0.025 |

**Table S9.** REE concentrations from the incubation solution, normalised to Post-Archean Australian Shale (PAAS). Normalised concentrations were calculated by dividing each (mean) original concentration by the corresponding PAAS value.

|  |  | **La** | **Ce** | **Pr** | **Nd** | **Sm** | **Eu** | **Gd** | **Tb** | **Dy** | **Y** | **Ho** | **Er** | **Yb** | **Lu** |
| --- | --- | --- | --- | --- | --- | --- | --- | --- | --- | --- | --- | --- | --- | --- | --- |
|  | PAAS values (10^-6^) | 44560 | 88250 | 10150 | 37320 | 6884 | 1215 | 6043 | 891.4 | 5325 | 27310 | 1053 | 3075 | 3012 | 438.6 |
| Post-Archean Australian Shale | **Site 1 - crust** |  |  |  |  |  |  |  |  |  |  |  |  |  |  |
| (PAAS) normalized data | 0 WEEK | 0.7 | 2.7 | 1.4 | 1.8 | 3.3 | 5.8 | 4.2 | 7.9 | 4.6 | 8.1 |  | 5.5 | 4.3 |  |
| (Pourmand et al.. 2012) | 15 WEEK | 0.4 | 0.2 | 0.5 | 0.6 | 1.2 | 4.1 | 1.6 | 5.6 | 4.6 | 10.8 |  | 23.2 | 56.6 | 85.1 |
|  | CTRL 0 | 0.5 | 2.9 | 0.9 | 1.4 | 2.0 | 4.1 | 2.3 |  | 2.6 | 5.6 |  | 3.3 | 2.3 |  |
|  | CTRL 15 | 0.3 | 0.2 | 0.4 | 0.6 | 1.3 |  | 1.8 |  | 8.5 | 22.6 | 18.0 | 43.3 | 84.3 | 111.7 |
|  |  |  |  |  |  |  |  |  |  |  |  |  |  |  |  |
|  | **Site 2 - discoidal** |  |  |  |  |  |  |  |  |  |  |  |  |  |  |
|  | 0 WEEK | 1.5 | 3.0 | 2.5 | 3.7 | 5.3 | 6.9 | 7.3 | 7.5 | 8.8 | 14.9 |  | 9.5 | 6.4 |  |
|  | 15 WEEK | 0.4 | 0.2 | 0.6 | 0.8 | 1.4 | 4.1 | 2.5 | 6.2 | 10.2 | 24.9 | 30.4 | 50.9 | 81.1 | 91.2 |
|  | CTRL 0 | 1.1 | 3.5 | 2.1 | 3.1 | 4.5 | 5.8 | 5.8 | 6.7 | 7.3 | 15.7 |  | 8.5 | 6.0 |  |
|  | CTRL 15 | 0.7 | 0.5 | 0.8 | 1.1 | 2.2 | 4.1 | 4.0 | 5.6 | 15.6 | 37.0 | 34.2 | 57.9 | 59.8 | 57.0 |
|  |  |  |  |  |  |  |  |  |  |  |  |  |  |  |  |
|  | **Site 3 - spheroidal** |  |  |  |  |  |  |  |  |  |  |  |  |  |  |
|  | 0 WEEK | 0.9 | 3.3 | 1.6 | 2.6 | 4.4 | 8.8 | 5.7 | 9.0 | 6.9 | 12.0 |  | 7.4 | 5.6 |  |
|  | 15 WEEK | 0.5 | 0.2 | 0.7 | 1.2 | 2.3 | 4.5 | 4.0 | 8.4 | 16.3 | 38.5 | 37.0 | 63.4 | 74.6 | 76.8 |
|  | CTRL 0 | 0.4 | 2.9 | 0.7 | 1.2 | 1.7 | 3.3 | 2.5 |  | 3.0 | 6.1 |  | 2.6 | 2.0 |  |
|  | CTRL 15 | 0.3 | 0.2 | 0.7 | 1.1 | 1.7 | 3.3 | 4.3 | 6.7 | 15.8 | 42.5 | 29.4 | 49.8 | 57.8 | 57.0 |

**Table S10.** Original REE concentrations (ppb) that were precipitated on bottle walls after 15 weeks of incubation from site 1 (crust), site 2 (discoidal), and site 3 (spheroidal). A-15 – C-15 = biotic triplicates at 15-week, CTRL-15 =abiotic control at 15-week.

|  |  |  |  | LREE |  |  |  | MREE |  |  |  | HREE |  |  |  |
| --- | --- | --- | --- | --- | --- | --- | --- | --- | --- | --- | --- | --- | --- | --- | --- |
|  |  | **La** | **Ce** | **Pr** | **Nd** | **Sm** | **Eu** | **Gd** | **Tb** | **Dy** | **Y** | **Ho** | **Er** | **Yb** | **Lu** |
|  |  | ppb | ppb | ppb | ppb | ppb | ppb | ppb | ppb | ppb | ppb | ppb | ppb | ppb | ppb |
| **Site 1 - crust** | 1A-15 | 29.10 | 64.38 | 7.47 | 28.08 | 5.61 | 0.08 | 4.13 | 0.35 | 0.96 | 3.01 | 0.09 | 0.23 | 0.10 | 0.01 |
|  | 1B-15 | 42.31 | 85.81 | 9.69 | 36.81 | 6.65 | 0.60 | 5.55 | 0.63 | 2.62 | 11.22 | 0.39 | 0.88 | 0.35 | 0.04 |
|  | 1C-15 | 8.90 | 17.91 | 2.01 | 7.52 | 1.49 | 0.16 | 1.37 | 0.19 | 0.90 | 4.57 | 0.14 | 0.36 | 0.24 | 0.03 |
|  | Mean | 26.77 | 56.03 | 6.39 | 24.14 | 4.58 | 0.28 | 3.69 | 0.39 | 1.49 | 6.26 | 0.21 | 0.49 | 0.23 | 0.03 |
|  | 1CTRL-15 | 7.19 | 14.99 | 1.74 | 6.73 | 1.31 | 0.10 | 1.10 | 0.12 | 0.42 | 1.72 | 0.05 | 0.13 | 0.07 | <0.008 |
|  |  |  |  |  |  |  |  |  |  |  |  |  |  |  |  |
| **Site 2 - discoidal** | 2A-15 | 16.47 | 34.49 | 3.90 | 14.21 | 2.99 | 0.08 | 2.42 | 0.27 | 0.98 | 3.64 | 0.12 | 0.25 | 0.12 | 0.01 |
|  | 2B-15 | 9.78 | 19.60 | 2.21 | 8.28 | 1.44 | 0.05 | 1.16 | 0.12 | 0.41 | 1.60 | 0.05 | 0.13 | 0.08 | <0.008 |
|  | 2C-15 | 103.47 | 203.28 | 22.57 | 83.25 | 15.59 | 0.63 | 12.42 | 1.27 | 4.53 | 16.33 | 0.56 | 1.19 | 0.45 | 0.05 |
|  | Mean | 43.24 | 85.79 | 9.56 | 35.24 | 6.67 | 0.25 | 5.33 | 0.55 | 1.97 | 7.19 | 0.24 | 0.52 | 0.21 | 0.03 |
|  | 2CTRL-15 | 11.89 | 24.39 | 2.44 | 8.96 | 1.46 | 0.09 | 1.22 | 0.13 | 0.58 | 2.83 | 0.09 | 0.23 | 0.15 | 0.02 |
|  |  |  |  |  |  |  |  |  |  |  |  |  |  |  |  |
| **Site 3 - spheroidal** | 3A-15 | 1.58 | 3.27 | 0.36 | 1.34 | 0.26 | 0.03 | 0.25 | 0.04 | 0.23 | 1.90 | 0.05 | 0.18 | 0.16 | 0.02 |
|  | 3B-15 | 4.44 | 10.17 | 0.96 | 3.74 | 0.66 | 0.08 | 0.63 | 0.07 | 0.37 | 2.34 | 0.06 | 0.19 | 0.14 | 0.01 |
|  | 3C-15 | 10.39 | 22.31 | 2.61 | 9.84 | 1.88 | 0.07 | 1.44 | 0.13 | 0.47 | 2.12 | 0.06 | 0.16 | 0.08 | 0.01 |
|  | Mean | 5.47 | 11.91 | 1.31 | 4.97 | 0.93 | 0.06 | 0.77 | 0.08 | 0.36 | 2.12 | 0.06 | 0.18 | 0.13 | 0.01 |
|  | 3 CTRL 15 | 1.64 | 2.94 | 0.37 | 1.39 | 0.27 | 0.04 | 0.24 | 0.03 | 0.17 | 1.13 | 0.03 | 0.09 | 0.06 | <0.008 |

**Table S11.** Normalised REE concentrations (ppb) that were precipitated on bottle walls after 15 weeks of incubation from site 1 (crust), site 2 (discoidal), and site 3 (spheroidal). Concentrations were normalised to Post-Archean Australian Shale (PAAS) by dividing each (mean) original concentration by the corresponding PAAS value.

|  | **La** | **Ce** | **Pr** | **Nd** | **Sm** | **Eu** | **Gd** | **Tb** | **Dy** | **Y** | **Ho** | **Er** | **Yb** | **Lu** |
| --- | --- | --- | --- | --- | --- | --- | --- | --- | --- | --- | --- | --- | --- | --- |
| PAAS values (10^-6)^ | 44560 | 88250 | 10150 | 37320 | 6884 | 1215 | 6043 | 891.4 | 5325 | 27310 | 1053 | 3075 | 3012 | 438.6 |
|  |  |  |  |  |  |  |  |  |  |  |  |  |  |  |
| Site 1 - 15 Precipitate | 601 | 635 | 630 | 647 | 665 | 229 | 610 | 432 | 280 | 229 | 199 | 159 | 77 | 59 |
| Site 1 - 15 Precipitate Control | 161 | 170 | 172 | 180 | 190 | 79 | 182 | 129 | 79 | 63 | 50 | 41 | 25 |  |
| Site 2 - 15 Precipitate | 970 | 972 | 942 | 944 | 970 | 205 | 882 | 618 | 371 | 263 | 231 | 170 | 71 | 68 |
| Site 2 - 15 Precipitate Control | 267 | 276 | 240 | 240 | 213 | 73 | 202 | 148 | 108 | 104 | 85 | 76 | 51 | 36 |
| Site 3 - 15 Precipitate | 123 | 135 | 129 | 133 | 135 | 50 | 128 | 90 | 67 | 78 | 53 | 57 | 42 | 30 |
| Site 3 - 15 Precipitate Control | 37 | 33 | 36 | 37 | 38 | 34 | 39 | 35 | 31 | 41 | 26 | 28 | 21 |  |

**Table S12.** Nitrate+nitrite, total nitrogen, and phosphate concentrations (µg/L) from the Artificial brackish seawater and from the incubation solution of microcosms in site 1 (crust), site 2 (discoidal), and site 3 (spheroidal). A-15 – C-15 =biotic triplicates at 15-week.

|  |  | (NO3+NO2)-N | TN | PO4-P |
| --- | --- | --- | --- | --- |
|  |  | µg/l | µg/l | µg/l |
|  | Artificial brackish seawater | <3 (1.8) | 3970 | 282 |
|  |  |  |  |  |
| **Site 1 (crust)** | 1. A-15 | 5093 | 5258 | 65 |
|  | 1. B-15 | 5073 | 5819 | 97 |
|  | 1. C-15 | 4695 | 5124 | 42 |
|  | Mean | 4954 | 5401 | 68 |
|  | std | 183 | 301 | 23 |
|  |  |  |  |  |
| **Site 2 (discoidal)** | 2. A-15 | 4837 | 4779 | 138 |
|  | 2. B-15 | 5210 | 5873 | 86 |
|  | 2. C-15 | 4772 | 5932 | 92 |
|  | Mean | 4940 | 5528 | 105 |
|  | std | 193 | 530 | 24 |
|  |  |  |  |  |
| **Site 3 (spheroidal)** | 3. A-15 | 3701 | 4135 | 160 |
|  | 3. B-15 | 3756 | 4586 | 188 |
|  | 3. C-15 | 4258 | 4729 | 162 |
|  | Mean | 3905 | 4483 | 170 |
|  | std | 250 | 253 | 13 |

**Table S13 (A).** Shannon diversity index (H′) values from fluffy, sediment, and Fe-Mn concretion samples collected immediately after sampling (intrinsic) in 2023. Samples are arranged based on collected environment.

| **2023** | | | | | |
| --- | --- | --- | --- | --- | --- |
| Sample name | Site | Type | Environment | Shannon | Mean_shannon (std) |
| D_2RNA | Site 2 (discoidal) | DNA | Concretion | 5.24 | 5.15 (± 0.18), n=12 |
| D_1RNA_23 | Site 1 (crust) | DNA | Concretion | 5.22 |  |
| D_3RNA_23 | Site 3 (spheroidal) | DNA | Concretion | 5.4 |  |
| R_1A_RNA_23 | Site 1 (crust) | RNA | Concretion | 4.97 |  |
| R_1B_RNA_23 | Site 1 (crust) | RNA | Concretion | 4.85 |  |
| R_1C_RNA_23 | Site 1 (crust) | RNA | Concretion | 5.01 |  |
| R_2A_RNA_23 | Site 2 (discoidal) | RNA | Concretion | 5.03 |  |
| R_2B_RNA_23 | Site 2 (discoidal) | RNA | Concretion | 5.15 |  |
| R_2C_RNA_23 | Site 2 (discoidal) | RNA | Concretion | 5.12 |  |
| R_3A_RNA_23 | Site 3 (spheroidal) | RNA | Concretion | 5.09 |  |
| R_3B_RNA_23 | Site 3 (spheroidal) | RNA | Concretion | 5.18 |  |
| R_3C_RNA_23 | Site 3 (spheroidal) | RNA | Concretion | 5.49 |  |
| D_2RNA_23 | Site 2 (discoidal) | DNA | Concretion | 5.28 |  |
| D_3FLUF_23 | Site 3 (spheroidal) | DNA | Fluffy | 4.42 | 4.67 (± 0.35), n=12 |
| R_1A_FLUF_23 | Site 1 (crust) | RNA | Fluffy | 5.36 |  |
| R_1B_FLUF_23 | Site 1 (crust) | RNA | Fluffy | 5.1 |  |
| R_1C_FLUF_23 | Site 1 (crust) | RNA | Fluffy | 4.76 |  |
| R_2A_FLUF_23 | Site 2 (discoidal) | RNA | Fluffy | 4.68 |  |
| R_2B_FLUF_23 | Site 2 (discoidal) | RNA | Fluffy | 4.46 |  |
| R_2C_FLUF_23 | Site 2 (discoidal) | RNA | Fluffy | 4.59 |  |
| R_3A_FLUF_23 | Site 3 (spheroidal) | RNA | Fluffy | 4.21 |  |
| R_3B_FLUF_23 | Site 3 (spheroidal) | RNA | Fluffy | 4.28 |  |
| R_3C_FLUF_23 | Site 3 (spheroidal) | RNA | Fluffy | 4.56 |  |
| D_1FLUF_23 | Site 1 (crust) | DNA | Fluffy | 5.09 |  |
| D_2FLUF_23 | Site 2 (discoidal) | DNA | Fluffy | 4.48 |  |
| D_1SED_23 | Site 1 (crust) | DNA | Sediment | 5.58 | 5.42 (± 0.55), n=7 |
| D_2SED_23 | Site 2 (discoidal) | DNA | Sediment | 5.76 |  |
| D_3SED_23 | Site 3 (spheroidal) | DNA | Sediment | 5.54 |  |
| R_1A_SED_23 | Site 1 (crust) | RNA | Sediment | 5.59 |  |
| R_1B_SED_23 | Site 1 (crust) | RNA | Sediment | 5.59 |  |
| R_3B_SED_23 | Site 3 (spheroidal) | RNA | Sediment | 5.68 |  |
| R_3C_SED_23 | Site 3 (spheroidal) | RNA | Sediment | 4.18 |  |

**Table S13 (B).** Shannon diversity index (H′) values from fluffy, sediment, and Fe-Mn concretion samples collected immediately after sampling (intrinsic) in 2022. Samples are arranged based on the collected environment.

| **2022** | | | | | |
| --- | --- | --- | --- | --- | --- |
| Sample name | Site | Type | Environment | Shannon | Mean_shannon (std) |
| D_1RNA | Site 1 (crust) | DNA | Concretion | 4.58 | 4.94 (± 0.37), n=6 |
| D_3RNA | Site 3 (spheroidal) | DNA | Concretion | 5.26 |  |
| R_1RNA | Site 1 (crust) | RNA | Concretion | 4.04 |  |
| R_2RNA | Site 2 (discoidal) | RNA | Concretion | 5.23 |  |
| R_3RNA | Site 3 (spheroidal) | RNA | Concretion | 4.91 |  |
| D_1FLUF | Site 1 (crust) | DNA | Fluffy | 4.86 | 4.21 (± 0.32), n=12 |
| D_2FLUF | Site 2 (discoidal) | DNA | Fluffy | 3.09 |  |
| D_3FLUF | Site 3 (spheroidal) | DNA | Fluffy | 4.28 |  |
| R_1A_FLUF | Site 1 (crust) | RNA | Fluffy | 4.48 |  |
| R_1B_FLUF | Site 1 (crust) | RNA | Fluffy | 4.02 |  |
| R_1C_FLUF | Site 1 (crust) | RNA | Fluffy | 4.58 |  |
| R_2A_FLUF | Site 2 (discoidal) | RNA | Fluffy | 4.22 |  |
| R_2B_FLUF | Site 2 (discoidal) | RNA | Fluffy | 3.78 |  |
| R_2C_FLUF | Site 2 (discoidal) | RNA | Fluffy | 3.79 |  |
| R_3A_FLUF | Site 3 (spheroidal) | RNA | Fluffy | 4.15 |  |
| R_3B_FLUF | Site 3 (spheroidal) | RNA | Fluffy | 4.19 |  |
| R_3C_FLUF | Site 3 (spheroidal) | RNA | Fluffy | 4.07 |  |
| D_1SED | Site 1 (crust) | DNA | Sediment | 5.53 | 5.59 (± 0.09), n=3 |
| D_2SED | Site 2 (discoidal) | DNA | Sediment | 5.53 |  |
| D_3SED | Site 3 (spheroidal) | DNA | Sediment | 5.69 |  |

**Table S14.** Shannon diversity index (H′) values from -Mn concretion samples collected from incubation experiments in 2023 and 2022. Samples are arranged based on the site.

**Table S15 (A).** ANOVA and Tukey HSD post-hoc comparisons of Shannon diversity between environments from 2023 and 2022. diff = difference in means, lwr and upr = confidence levels, adj. p = adjusted p-values for all possible pairs.

| **2023** | | | | |
| --- | --- | --- | --- | --- |
| comparison | diff | lwr | upr | p adj |
| Fluffy (2023)-Concretion (2023) | -0.483 | -0.843 | -0.124 | 0.007 |
| Sediment (2023)-Concretion (2023) | 0.268 | -0.151 | 0.687 | 0.27 |
| Sediment (2023)-Fluffy (2023) | 0.751 | 0.332 | 1.17 | <0.001 |

| **2022** | | | | |
| --- | --- | --- | --- | --- |
| comparison | diff | lwr | upr | p adj |
| Fluffy (2022)-Concretion (2022) | -0.728 | -1.136 | -0.321 | 0.001 |
| Sediment (2022)-Concretion (2022) | 0.647 | 0.071 | 1.223 | 0.026 |
| Sediment (2022)-Fluffy (2022) | 1.375 | 0.849 | 1.901 | <0.001 |

**Table S15 (B).** ANOVA and Tukey HSD post-hoc comparisons of Shannon diversity between morphotypes from incubation experiment in 2023. Welch’s ANOVA, followed by Games-Howell post hoc comparisons were done from 2022 experiment. diff = difference in means, lwr and upr = confidence levels, adj. p = adjusted p-values for all possible pairs.

| **2023** | | | | |
| --- | --- | --- | --- | --- |
|  | diff | lwr | upr | p adj |
| Site 2 (discoidal)-Site 1 (crust) | 0.094 | -0.051 | 0.24 | 0.264 |
| Site 3 (spheroidal)-Site 1 (crust) | 0.29 | 0.145 | 0.435 | <0.001 |
| Site 3 (spheroidal)-Site 2 (discoidal) | 0.196 | 0.05 | 0.341 | 0.006 |

| **2022** | | | | |
| --- | --- | --- | --- | --- |
|  | diff | lwr | upr | p adj |
| Site 2 (discoidal)-Site 1 (crust) | 0.654 | 0.406 | 0.902 | <0.001 |
| Site 3 (spheroidal)-Site 1 (crust) | 0.736 | 0.485 | 0.988 | <0.001 |
| Site 3 (spheroidal)-Site 2 (discoidal) | 0.083 | -0.019 | 0.185 | 0.125 |

**Table S16**. PERMANOVA results based on Bray-Curtis distances from intrinsic fluffy, sediment, and Fe-Mn concretion samples in 2023 and 2022. P_adj_BH = Benjamini–Hochberg.

| **2023** | | | |
| --- | --- | --- | --- |
| Comparison | R2 | p_value | p_adj_BH |
| Concretion vs. Fluffy | 0.66 | 0.001 | 0.001 |
| Concretion vs. Sediment | 0.54 | 0.001 | 0.001 |
| Fluffy vs. Sediment | 0.46 | 0.001 | 0.001 |

| **2022** | | | |
| --- | --- | --- | --- |
| Comparison | R2 | p_value | p_adj_BH |
| Concretion vs. Fluffy | 0.65 | 0.001 | 0.003 |
| Concretion vs. Sediment | 0.41 | 0.014 | 0.014 |
| Fluffy vs. Sediment | 0.55 | 0.003 | 0.005 |

**Table S17.** PERMANOVA results based on Bray-Curtis distances from Fe-Mn concretion samples collected from incubation experiments and envfit results from each element. Elements are arranged based on their adjusted p-value. P_adj_BH = Benjamini–Hochberg.
